# Loss of species and genetic diversity during colonization: Insights from acanthocephalan parasites in northern European seals

**DOI:** 10.1002/ece3.10608

**Published:** 2023-10-19

**Authors:** Ludmila Sromek, Eeva Ylinen, Mervi Kunnasranta, Simo N. Maduna, Tuula Sinisalo, Craig T. Michell, Kit M. Kovacs, Christian Lydersen, Evgeny Ieshko, Elena Andrievskaya, Vyacheslav Alexeev, Sonja Leidenberger, Snorre B. Hagen, Tommi Nyman

**Affiliations:** ^1^ Department of Marine Ecosystems Functioning, Institute of Oceanography University of Gdansk Gdynia Poland; ^2^ Department of Environmental and Biological Sciences University of Eastern Finland Joensuu Finland; ^3^ Natural Resources Institute Finland Joensuu Finland; ^4^ Department of Ecosystem in the Barents Region Norwegian Institute of Bioeconomy Research Svanvik Norway; ^5^ Department of Biological and Environmental Sciences University of Jyväskylä Jyväskylä Finland; ^6^ Red Sea Research Center King Abdullah University of Science and Technology Jeddah Saudi Arabia; ^7^ Norwegian Polar Institute, Fram Centre Tromsø Norway; ^8^ Institute of Biology, Karelian Research Centre Russian Academy of Sciences Petrozavodsk Russia; ^9^ The Baltic Ringed Seal Foundation St. Petersburg Russia; ^10^ Department of Biology and Bioinformatics, School of Bioscience University of Skövde Skövde Sweden

**Keywords:** Acanthocephala, genetic diversity, phylogeography, population bottlenecks, population genomics, seal parasites

## Abstract

Studies on host–parasite systems that have experienced distributional shifts, range fragmentation, and population declines in the past can provide information regarding how parasite community richness and genetic diversity will change as a result of anthropogenic environmental changes in the future. Here, we studied how sequential postglacial colonization, shifts in habitat, and reduced host population sizes have influenced species richness and genetic diversity of *Corynosoma* (Acanthocephala: Polymorphidae) parasites in northern European marine, brackish, and freshwater seal populations. We collected *Corynosoma* population samples from Arctic, Baltic, Ladoga, and Saimaa ringed seal subspecies and Baltic gray seals, and then applied COI barcoding and triple‐enzyme restriction‐site associated DNA (3RAD) sequencing to delimit species, clarify their distributions and community structures, and elucidate patterns of intraspecific gene flow and genetic diversity. Our results showed that *Corynosoma* species diversity reflected host colonization histories and population sizes, with four species being present in the Arctic, three in the Baltic Sea, two in Lake Ladoga, and only one in Lake Saimaa. We found statistically significant population‐genetic differentiation within all three *Corynosoma* species that occur in more than one seal (sub)species. Genetic diversity tended to be high in *Corynosoma* populations originating from Arctic ringed seals and low in the landlocked populations. Our results indicate that acanthocephalan communities in landlocked seal populations are impoverished with respect to both species and intraspecific genetic diversity. Interestingly, the loss of genetic diversity within *Corynosoma* species seems to have been less drastic than in their seal hosts, possibly due to their large local effective population sizes resulting from high infection intensities and effective intra‐host population mixing. Our study highlights the utility of genomic methods in investigations of community composition and genetic diversity of understudied parasites.

## Graphical abstract

Acanthocephalan parasite communities in northern European landlocked seal populations are impoverished with respect to both species and intraspecific genetic diversity. Interestingly, the loss of genetic diversity within *Corynosoma* species seems to have been less drastic than in their seal hosts, possibly due to their large local effective population sizes resulting from high infection intensities and effective intra‐host population mixing. Our study highlights the utility of the 3RAD method in investigations of community composition and genetic diversity of understudied parasites.

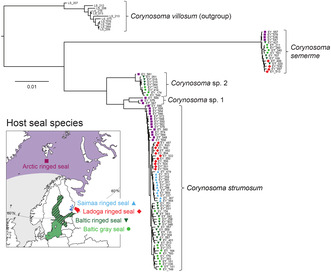

## 1 INTRODUCTION

Colonization of new areas and environments often leads to profound changes in host–parasite associations (Hoberg & Brooks, 2008, 2015; Nazarizadeh et al., 2023). Some parasites present in a source population may not survive in new environments, for example, due to a lack of suitable intermediate hosts (Hoberg & Brooks, 2008). Species may also be lost during colonization due to stochastic effects, including absence of parasites in the colonizing hosts or reduced transmission caused by low density of the founding host population (Dobson, 1988; Lloyd‐Smith et al., 2005; Mlynarek et al., 2017; Torchin et al., 2003). Such effects are well documented in birds inhabiting oceanic islands, which are characterized by a reduced number of parasite species compared to their mainland counterparts (Loiseau et al., 2017; Sari et al., 2013; Spurgin et al., 2012). For example, the haemosporidian parasite assemblage of Macaronesian blackcaps contains only about 10% of the species found on the continent (Pérez‐Rodríguez et al., 2013). Analogously, Louizi et al. (2023) found depauperate communities of monogenean gill parasites in cichlid fish occurring at the edge of their distribution range in northern Africa. On the other hand, hosts colonizing new areas may also acquire local species of parasites, giving rise to novel host–parasite associations (Hoberg & Brooks, 2008). An illuminating example of such turnover is provided by invasive Ponto‐Caspian gobies, in which the loss of native parasite species has been partly balanced off by acquisition of new parasites in their non‐native ranges (Kvach & Ondračková, 2020).

Genetic changes in parasites that establish with their host in a new area are less well understood. Generally, spatial population‐genetic structuring within parasite species is expected to reflect that of their hosts (Koop et al., 2014; Nieberding & Olivieri, 2007; Whiteman & Parker, 2005), and host population bottlenecks during colonization are expected to result in loss of genetic variability in parasites as well (Demastes et al., 2019; Nieberding et al., 2006; Thys et al., 2022). However, the extent of spatial differentiation and the severity of genetic erosion can differ markedly between hosts and parasites (Blakeslee et al., 2020; McCoy et al., 2005; Whiteman et al., 2007). The direction and magnitude of the differences will depend on the effective population size (*N*_e_) and the degree of isolation from other populations, both of which are shaped by host and parasite life history traits (Cole & Viney, 2018; Criscione & Blouin, 2005; Doña & Johnson, 2023; Huyse et al., 2005; van Schaik et al., 2015). In parasites with low host specificity or a complex life cycle involving a free‐living phase or mobile intermediate hosts, genetic structuring resulting from colonization can be quickly erased by dispersal (Jones & Britten, 2010; Mazé‐Guilmo et al., 2016). In contrast, if the barrier to gene flow is effective for both the host and its parasites, the parasite populations are expected to undergo faster genetic differentiation due to their shorter generation times (Nieberding et al., 2004; Virrueta Herrera et al., 2022; Whiteman & Parker, 2005). However, especially in the case of large‐bodied, long‐lived host species in which a single individual can support many parasite individuals, the effective population size of parasites can be larger than that of the hosts, making the parasites more resistant against genetic erosion (Huyse et al., 2005). Understanding the relative importance of these factors and how they interact with each other is important for conservation, as low genetic diversity of hosts often correlates with high parasite loads at both individual (Coltman et al., 1999; Hoffman et al., 2014) and population (Ekroth et al., 2019) levels.

**FIGURE 1:**
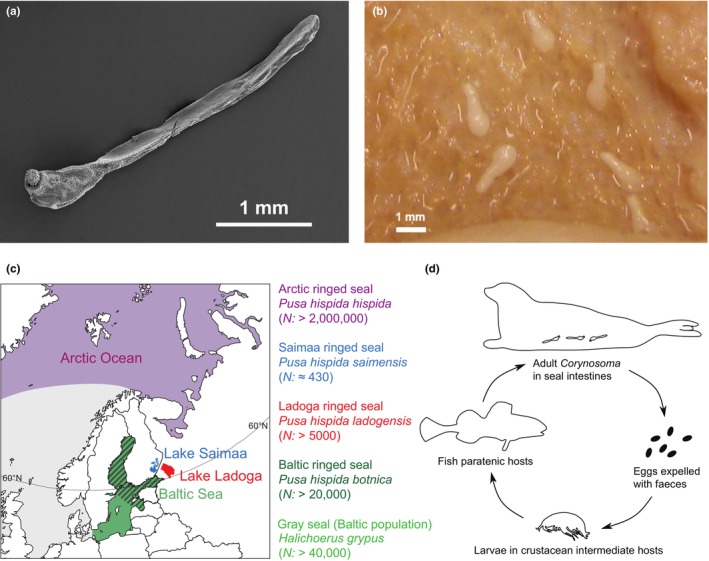
(a) SEM micrograph of a female *Corynosoma strumosum* collected from a gray seal from the Baltic Sea. (b) Specimens of *C. semerme* inside the large intestine of a gray seal. (c) Geographical distributions and estimated current population sizes of the seal populations from which *Corynosoma* parasites were collected for the present study. (d) Schematic life cycle of *Corynosoma* species associated with seals.

Here, we investigated how host population history and size influence community composition, population‐genetic structure, and genetic diversity of thorny‐headed worms belonging to the genus *Corynosoma* Lühe, 1904 (Acanthocephala: Polymorphidae) in ringed and gray seals inhabiting marine, brackish, and freshwater environments in northern Europe (Figure 1). Marine seals generally support diverse parasite communities consisting of many taxonomic groups, including acanthocephalans, nematodes, cestodes, trematodes, and arthropods (Leidenberger et al., 2007, 2020; Reckendorf et al., 2019; Walden et al., 2020). In contrast to several other parasite taxa, *Corynosoma* were able to adapt to brackish and freshwater environments when ringed seals colonized the Baltic Sea basin and large postglacial lakes that were formed at the end of the Pleistocene. *Corynosoma* are small to medium‐sized intestinal worms (typically 2–15 mm) with complex life cycles involving crustaceans (amphipods or isopods) as intermediate hosts, teleost fish as paratenic hosts, and marine mammals and seabirds as final hosts (Figure 1d) (Aznar et al., 2016; García‐Varela & de León, 2015; Leidenberger et al., 2020). While mild *Corynosoma* infections are asymptomatic, heavy infestations can cause intestinal inflammations, colonic ulcers, and tunica muscularis hypertrophy (Lakemeyer et al., 2020; Siebert et al., 2007).

Our study system comprises several closely related seal hosts with highly divergent population sizes, colonization histories, and degrees of isolation (Figure 1c). The Baltic ringed seal (*Pusa hispida botnica*), the Ladoga ringed seal (*P. h. ladogensis*), and the Saimaa ringed seal (*P. h. saimensis*) are all derived from Arctic ringed seals (*P. h. hispida*) that colonized the current Baltic Sea basin after the last glacial period (Ukkonen et al., 2014). Due to progressive land uplift, parts of the Baltic ringed seal population were trapped in Lake Saimaa c. 9000 years ago and in Lake Ladoga c. 5000 years ago (Saarnisto, 2011). The population estimates of these various ringed seal subspecies differ by four orders of magnitude (Figure 1c). With a population of several million individuals (Laidre et al., 2015; Reeves, 1998), the Arctic ringed seal is one of the most abundant marine mammals on Earth. By contrast, the Baltic ringed seal population currently numbers c. 20,000 individuals (Halkka & Tolvanen, 2017; HELCOM, 2018). The two freshwater populations are substantially smaller. The Ladoga ringed seal is classified as “Vulnerable” on the IUCN Red List (IUCN, 2022), and currently numbers c. 5000 individuals (Trukhanova et al., 2013), and the Saimaa ringed seal is classified as “Endangered” due to its population size of approximately 400 individuals (IUCN, 2022; Kunnasranta et al., 2021).

The genetic diversity of the four focal ringed seal subspecies reflects their population sizes and colonization history. The numerous Arctic ringed seal is exceptionally diverse genetically in comparison to other seals (Peart et al., 2020; Stoffel et al., 2018). Genetic variability remains nearly as high in the Baltic ringed seal, despite a marked population reduction in the 20th century due to intensive hunting and environmental pollution (Löytynoja et al., 2023; Nyman et al., 2014; Palo et al., 2001). However, the isolated Saimaa ringed seal has lost about half of the genetic diversity present in its Baltic ancestors (Kunnasranta et al., 2021; Löytynoja et al., 2023; Nyman et al., 2014; Stoffel et al., 2018). Genetic erosion has been less severe in the Ladoga ringed seal, probably due to a higher number of colonizers and a less dramatic 20th‐century anthropogenically induced population bottleneck (Löytynoja et al., 2023; Nyman et al., 2014).

Interestingly, in the lakes, ringed seals constitute the only definitive hosts available for *Corynosoma* parasites, while the Baltic Sea is also inhabited by gray seals (*Halichoerus grypus*) (over 40,000 individuals; Scharff‐Olsen et al., 2019) and harbor seals (*Phoca vitulina*) (over 1000 individuals; Blanchet et al., 2021). The spectrum of potential *Corynosoma* hosts is even wider in the Arctic, where the ringed seal occurs with several other species of true seals (Hamilton et al., 2022). This provides a unique opportunity to trace how the availability of other host species affects the diversity of parasite communities.

Based on morphological studies, the Arctic ringed seal is believed to be the definitive host for at least six *Corynosoma* species (Kelly et al., 2010), of which only one to three have been reported from the Baltic Sea and the two freshwater populations (Delyamure et al., 1980; Leidenberger et al., 2020; Sinisalo et al., 2003; Valtonen et al., 2004). However, these traditional views of *Corynosoma* community diversity remain to be confirmed, because morphological identification of *Corynosoma* specimens has proven to be very challenging due to their reduced and variable morphology (Aznar et al., 2006; Leidenberger et al., 2019; Nickol et al., 2002). For example, in the Baltic Sea and lakes Ladoga and Saimaa, the traditional broad concept of *C. strumosum* was in the early 2000s gradually replaced by a division into *C. magdaleni* and a more narrowly defined *C. strumosum* (Leidenberger et al., 2020; Nickol et al., 2002; Sinisalo, 2007; Valtonen et al., 2004). Furthermore, a recent survey of *Corynosoma* communities in harbor and gray seals from the North Sea and Baltic Sea by Waindok et al. (2018) showed that none of the 35 specimens morphologically determined as *C. strumosum* belonged to this species according to their mitochondrial cytochrome *c* oxidase subunit 1 (COI) gene sequences and nuclear ribosomal internal transcribed spacer (ITS) sequences. Instead, the molecular markers indicated that 32 individuals should be assigned to *C. magdaleni*, while the remaining three specimens represented a possibly undescribed cryptic species, “Candidatus *Corynosoma nortmeri*.” Genetic studies from other parts of the world have likewise found evidence of both over‐ (Lisitsyna et al., 2019; Sasaki et al., 2019) and under‐splitting (Hernández‐Orts et al., 2022) of *Corynosoma* lineages. With all of this in mind, supplementing morphological investigations with detailed molecular‐genetic analyses seem to be essential for determining the true species diversity and community structures of acanthocephalan parasites infecting seals.

The main aims of our study were to: (1) infer whether community composition and species diversity of *Corynosoma* parasites reflect the sequential colonization histories and current population sizes of their seal hosts, (2) examine whether widely distributed *Corynosoma* species exhibit intraspecific genetic structuring with respect to seal host (sub)species or geographic areas, and (3) infer whether intraspecific genetic diversity in the parasites reflects the widely differing levels of genetic variability found in their seal hosts. To these ends, we first sequenced the standard “DNA barcode” section of the mitochondrial COI gene from 578 *Corynosoma* individuals collected from Arctic, Baltic, Ladoga, and Saimaa subspecies of ringed seal and Baltic gray seals, and then confirmed our barcode‐based species delimitations by performing restriction‐site associated DNA sequencing (RADseq) on a subset of individuals representing different COI clades. After species delimitation, we inferred *Corynosoma* community composition in the focal seal (sub)species and estimated the levels of population‐genetic differentiation among, and genetic diversity within, the different host populations and geographic areas. Based on the sequence of postglacial colonization and current population sizes of the focal seal (sub)species, we hypothesized that: (i) *Corynosoma* community richness should decrease from the Arctic toward the Baltic Sea and the two small and isolated landlocked populations, (ii) widespread *Corynosoma* species should exhibit population‐genetic differentiation across geographic areas and host (sub)species, and (iii) intraspecific genetic diversity should decrease along with *Corynosoma* community richness, and should reflect the levels found in the seal hosts.

## 2 METHODS

### 2.1 Sample collection

The 578 adult acanthocephalan worms studied herein were collected during necropsies of the digestive tracts of 18 Arctic ringed seals, 12 Baltic ringed seals, 25 Saimaa ringed seals, 4 Ladoga ringed seals, and 18 Baltic gray seals (see Appendix S1). Seals were either found dead (stranded or by‐caught individuals) (Saimaa and Ladoga) or sampled for research purposes (Baltic) as part of seal health monitoring programs of the University of Eastern Finland and Natural Resources Institute Finland (permits MMM 234/400/2008 and VARELY/3480/2016), and the Baltic Ringed Seal Foundation in Russia. The seals from the Arctic were collected during the regular sport hunting that takes place each year in Svalbard.

To investigate the spatial distribution of different *Corynosoma* species along the gastrointestinal tracts (see Appendix S2), the intestines were divided into 10 equal‐length sections of the small intestine (SI 1–10), the cecum (CE), and two equal‐length parts of the large intestine (LI 1 and 2). Specimens from each seal and intestinal section were collected into separate 2‐mL screw‐cap tubes with 99.5% ethanol and stored at −20°C until analysis. This was done for all seals, except the ringed seals from the Arctic and two individuals from Ladoga, for which the digestive tract was divided into only the small and large intestines. Generally, one *Corynosoma* individual per intestinal section of each individual seal was selected for DNA extraction and genetic analysis, but several individuals per section were processed if some sections lacked parasites. In addition to the focal seal species and populations, we sampled 11 juvenile acanthocephalans from two bearded seals (*Erignathus barbatus*) from Svalbard to serve as an outgroup in phylogenetic analyses based on RADseq data (Appendix S1: Table S1.1).

We evaluated the concordance between traditional morphology‐based taxonomy and molecular species delimitation by randomly selecting 55 *Corynosoma* individuals collected from Saimaa and Baltic ringed and gray seals for morphological blind‐test identification (Appendix S2). These specimens were pre‐identified under a stereomicroscope and then processed in a manner similar to all of the other samples.

### 2.2 DNA extraction

Genomic DNA was extracted from the tail end of sampled individuals using Qiagen DNeasy Tissue Kits (Qiagen) in accordance with the manufacturer's instructions. Specimens from bearded seals were extracted using the bead‐based BOMB DNA extraction protocol (Oberacker et al., 2019). DNA concentrations of extracts used for RADseq (see below) were quantified using a Quantus Fluorometer (Promega).

### 2.3 COI barcode and RADseq datasets

The standard DNA barcode region of the mitochondrial COI gene (Hebert et al., 2003) was PCR amplified and sequenced in both directions using a newly developed set of primers that—in contrast to previous protocols—allows sequencing of the whole 655 bp barcode region in *Corynosoma* (see Appendix S3). Following alignment with MUSCLE implemented in MEGA‐X (Kumar et al., 2018), the COI sequences were translated to confirm the absence of premature stop codons or frameshifts indicative of sequencing errors. The full COI barcode dataset consisted of an alignment of 655 bp from 578 *Corynosoma* individuals.

Based on the results of the species delimitation analyses based on the COI sequence dataset (see below), we selected a subset of the barcoded individuals for RAD sequencing as follows: from 10 to 14 individuals of *C. strumosum*, 4 individuals of *C. semerme*, and up to 4 individuals of *Corynosoma* sp. 1 and *Corynosoma* sp. 2 were randomly sampled per seal (sub)species from those extracts having a concentration above 1 ng/μl. In addition to this random sampling, we included 3 additional individuals based on their potentially interesting positions on the COI barcode NJ tree (Appendix S3: Figure S3.1A).

Due to the small size of *Corynosoma* worms, we used the novel 3RAD approach of Bayona‐Vásquez et al. (2019), which, compared to previous ddRAD protocols, does not require a high starting DNA concentration. Two independently indexed libraries were prepared using ClaI, MspI, and BamHI HF restriction enzymes (see Appendix S4). After sequencing, de‐multiplexed reads from two libraries were pooled and assembled de novo using ipyrad v. 0.9.57 (Eaton & Overcast, 2020). Prior to the final assembly, an exploratory analysis with eight individuals that were replicated in both libraries was performed to select the clustering threshold of 90%, which maximized the number of loci and SNPs recovered at low error rates (Appendix S4). The assembled set of loci was filtered to ensure locus sharing across COI barcode clusters and to remove potential contaminant loci arising from the seal host DNA (Appendix S4). The 3RAD sequencing and subsequent clustering and filtering steps resulted in a dataset containing 1005 RAD loci with 24,451 SNPs (Appendix S4: Table S4.1); this full dataset was then filtered for the separate analyses below (Appendix S4: Figure S4.3).

### 2.4 Species delimitation

A neighbor‐joining (NJ) tree for the 578 COI sequences was constructed in MEGA‐X based on Kimura's (1980) two‐parameter (K2P) model, with group support estimated using 500 bootstrap replicates. Next, an analysis using the distance‐based automatic barcode gap discovery (ABGD; Puillandre et al., 2012) species delimitation method was undertaken on the ABGD web interface (https://bioinfo.mnhn.fr/abi/public/abgd/abgdweb.html) based on the K2P model. We then queried the barcodes against previously published *Corynosoma* sequences on GenBank using the BLAST search tool and assigned our COI barcode sequences to species based on their top match statistic (i.e., highest percent identity), position on the NJ tree, and ABGD results.

We first assessed overall genetic structuring in our RADseq dataset using the model‐based clustering approach implemented in RADpainter and fineRADstructure (Malinsky et al., 2018). This method groups together individuals with high levels of shared co‐ancestry based on haplotype relationships (Malinsky et al., 2018). To get the haplotype matrix from the ipyrad output, we used the script *finerad_input.py* written by E. M. Ortiz and available via https://github.com/edgardomortiz/fineRADstructure‐tools. In the fineRADstructure clustering algorithm, 100,000 Markov chain iterations were used, with a burn‐in of 100,000 iterations and with sampling occurring every 1000 iterations. Next, the SNP data (.vcf file format from ipyrad) were imported into R v. 4.0.2 (R Core Team, 2022) using the vcfR v. 1.12.0 package (Knaus & Grünwald, 2017) and analyzed in the adegenet v. 2.1.3 package (Jombart, 2008). To assess groupings among individuals based on the SNP data, we used model‐free discriminant analysis of principal components (DAPC) with a priori group designations based on mitochondrial DNA haplogroups. To assess how many principal components (PCs) to retain, we used cross‐validation (*xvalDapc* function) and retained the number of PCs with the lowest mean squared error. Finally, we constructed a maximum‐likelihood (ML) phylogeny based on the concatenated RAD loci in IQ‐TREE v. 2.2.0 (Minh et al., 2020). The analysis implemented an edge‐linked partition model and 1000 ultrafast bootstrap replicates. The resultant phylogenetic tree was rooted by using *C. villosum* specimens sampled from bearded seal as an outgroup (Appendix S3).

### 2.5 Estimation of genetic differentiation across hosts and geographical areas

To visualize relationships among *Corynosoma* COI haplotypes and their frequencies in different seal (sub)species and geographic areas, we constructed a TCS haplotype network (Clement et al., 2002) in PopART v. 1.7 (Leigh & Bryant, 2015). For this analysis, 44 COI sequences shorter than 620 bp were excluded, and an alignment with 623 nucleotide sites was analyzed. To gain insight into the diversity of haplotypes on a wider geographical scale, we repeated this analysis by adding 61 *Corynosoma* sequences retrieved from GenBank (Appendix S3: Table S3.1). The GenBank sequences (accessed 31 March 2022) were retrieved using Entrez query *Corynosoma* [Organism] and manually selected to include all *C. strumosum*, *C. magdaleni*, *C. semerme*, and “Candidatus *Corynosoma nortmeri*” COI sequences that were longer than 600 bp. The alignment used in this expanded analysis included 601 nucleotide sites.

We tested for the presence of intraspecific population‐genetic structuring in *Corynosoma* COI barcode variation across host (sub)species and geographical areas by estimating overall and between‐population Φ_ST_ values in Arlequin v. 3.5.2.2 (Excoffier & Lischer, 2010) based on K2P distances among haplotypes. The calculations of overall differentiation were done using the locus‐by‐locus mode, and for both overall and pairwise differentiation the site‐specific maximum proportion of missing data was set to 0.05. Statistical significance of parameter estimates was determined through 10,000 randomizations of individual haplotypes across host (sub)species.

To produce an overview of intraspecific genetic differentiation across *Corynosoma* populations collected from different hosts and areas, we first plotted the origin of each specimen to the aforementioned fineRADstructure co‐ancestry matrix and the ML tree. For statistical analyses of intraspecific genetic differentiation and diversity, we constructed species‐specific SNP matrices for the four *Corynosoma* species present in our dataset and re‐filtered each matrix to include only variable, bi‐allelic sites that occurred in at least 50% of the individuals from each host (sub)species (Appendix S4: Figure S4.3). We tested for the presence of host‐associated genetic structure within *C. strumosum*, *C. semerme*, and *Corynosoma* sp. 2 by estimating overall and pairwise *F*_ST_ values (Weir & Cockerham, 1984) across host (sub)species in the hierfstat v. 0.5.11 R package (Goudet & Jombart, 2022). The 95% confidence intervals of the estimates were computed using 10,000 bootstrap replicates. To test for the presence of correlated differentiation in the COI and RADseq datasets within *C. strumosum* and *C. semerme*, we calculated Pearson correlation coefficients for between‐population estimates and inferred statistical significance based on two‐tailed Mantel tests with 10,000 permutations in XLSTAT v. 2023.14.4.

Finally, we performed a more detailed analysis of intraspecific genetic structuring within *C. strumosum*, which was present in all examined seal populations. For this, we used Admixture analysis in the LEA v. 3.8.0 R package (Frichot & François, 2015) using the aforementioned species‐specific SNP matrix, which was subsampled to one, randomly selected SNP per RAD locus to avoid tight linkage among loci (Appendix S4: Figure S4.3). We performed 10 replicate runs for each number of ancestral populations (*K*) ranging from 1 to 10 and chose the value of *K* for which the cross‐entropy criterion was the lowest.

### 2.6 Estimation of genetic diversity

To estimate population‐specific diversity in the full mitochondrial COI barcode dataset, we used Arlequin to estimate standard diversity indices (number of haplotypes, gene diversity, and nucleotide diversity) for the population samples of each *Corynosoma* species collected from each seal (sub)species. Haplotypes were inferred from distance matrices, nucleotide diversity was estimated based on K2P distances among haplotypes, and the site‐specific maximum proportion of missing data was set to 0.05.

To obtain corresponding estimates for the RADseq data, we used the four species‐level datasets (Appendix S4: Figure S4.3) to estimate and compare intraspecific nuclear genetic diversity in population samples collected from different host (sub)species. For this, we calculated expected heterozygosity across loci for each population and tested between‐population differences using pairwise Wilcoxon signed‐rank tests (in which estimates at loci represented pairs).

## 3 RESULTS

### 3.1 Species delimitation

When applying a prior maximal intraspecific divergence of 0.01, the ABGD method placed the barcode gap at a distance of 0.019 and indicated the presence of four species of *Corynosoma* in the full COI barcode dataset (Appendix S3: Figure S3.1A,B). Based on the highest percent identity to GenBank sequences, we assigned the samples to *C. strumosum, C. semerme*, *Corynosoma* sp. 1, and *Corynosoma* sp. 2 (Appendix S3). Within *C. strumosum*, a cluster of 221 barcode sequences that had the highest percent identity (98.6%–100%) with a published sequence of *C. magdaleni* (GenBank acc. no. EF467872) could be distinguished on the NJ tree (Appendix S3: Figure S3.1A). However, this group of sequences was not delimited as a separate species by the ABGD method even at lower values of maximal intraspecific divergence. The morphological blind test likewise indicated that *C*. “*magdaleni*” cannot be reliably separated from *C. strumosum* (Appendix S2).

**FIGURE 2:**
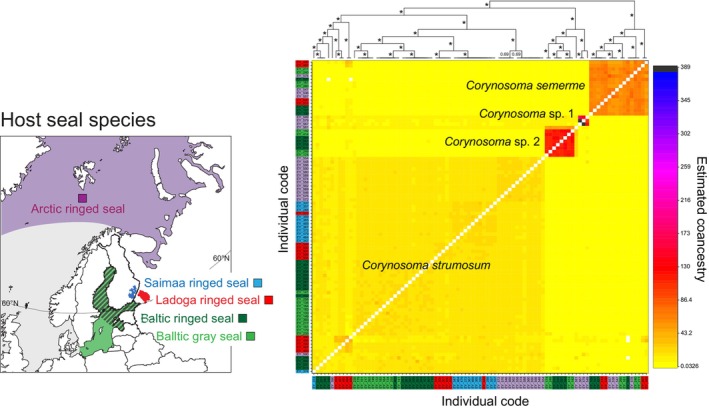
FineRADstructure plot derived from haplotype data of 1005 RADseq loci from 91 *Corynosoma* individuals collected from four northern European subspecies of ringed seal and Baltic gray seals. The heat map depicts pairwise co‐ancestry among individuals according to the color scale bar shown to the right of the matrix. Values next to the branches of the tree above the plot are posterior assignment probabilities (asterisks denote probability of 1.00). Individual codes are colored according to the seal host (sub)species from which the individual was collected (see inset map).

**FIGURE 3:**
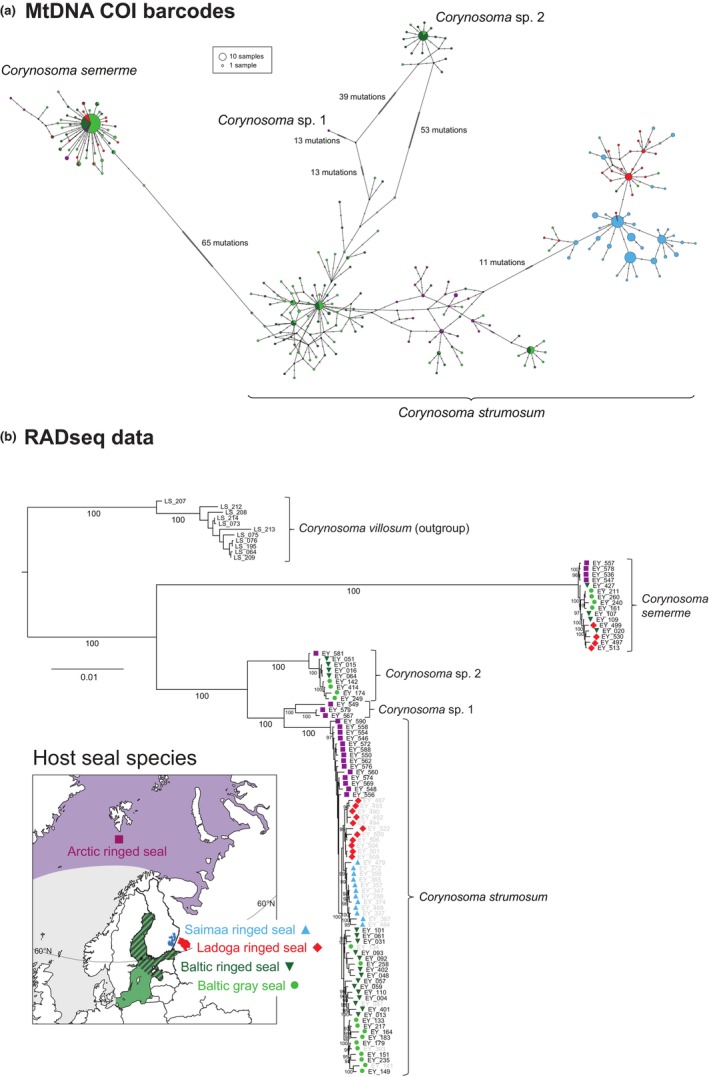
(a) TCS haplotype network of *Corynosoma* COI barcode sequences (*N* = 534). Circle and section colors denote seal host (sub)species and geographical areas (see inset map), while the size of the circles is proportional to the number of haplotypes (see legend). Tick marks along branches denote mutational steps. (For the results of an analysis including also *Corynosoma* reference sequences retrieved from GenBank, see Appendix S3: Figure S3.2). (b) Maximum‐likelihood tree for 102 *Corynosoma* individuals based on concatenated RADseq loci. Branch lengths are proportional to the number of substitutions per site, numbers below branches are ultrafast bootstrap support values (only values ≥95% shown). Colored symbols next to specimen labels indicate the seal host (sub)species and geographical area from which each individual was sampled (see inset map). *Corynosoma strumosum* individuals belonging to the *C*. “*magdaleni*” clade in the COI barcode NJ tree (Appendix S3: Figure S3.1) are indicated by gray specimen labels.

Our haplotype‐based fineRADstructure plot (Figure 2) revealed four co‐ancestry groups that corresponded to the *Corynosoma* species delimited based on mitochondrial COI barcode sequences. The SNP‐based DAPC analysis produced similar results, with the exception of a single individual of *Corynosoma* sp. 2 that was grouped within a cluster formed by intermixed *C. strumosum* and *C*. “*magdaleni*” individuals (Appendix S4: Figure S4.4). The four species‐level clades were, however, strongly supported by the ML tree estimated based on concatenated RADseq loci (Figure 3b). Notably, the individuals belonging to the *C*. “*magdaleni*” cluster in the COI NJ tree (Appendix S3: Figure S3.1A) did not form a monophyletic group in the ML tree (gray specimen labels on Figure 3b), and the DAPC analysis (Appendix S4: Figure S4.4) confirmed that specimens belonging to the *C*. “*magdaleni*” COI clade cannot be reliably distinguished from *C. strumosum* based on their multilocus genotypes.

### 3.2 Community structure and genetic differentiation

*Corynosoma strumosum* was the most widely distributed species being found in all studied seal (sub)species (Figure 3 and Appendix S3: Figure S3.1A). *Corynosoma semerme* was found in all host populations except the Saimaa ringed seal. The other two acanthocephalan species had more restricted geographical distributions: *Corynosoma* sp. 1 was present only in the Arctic ringed seal, while *Corynosoma* sp. 2 was found in Arctic and Baltic ringed seals and Baltic gray seals (Figures 3 and Appendix S3: Figure S3.1A).

The TCS haplotype network showed clear signs of host‐associated and geographical variation within the widely distributed *C. strumosum* (Figure 3a). For this species, differentiation in haplotype frequencies was particularly evident between lakes Saimaa and Ladoga, and between the samples collected from Baltic and Arctic seal populations. In contrast, the same common haplotypes representing *C. strumosum*, *C. semerme*, and *Corynosoma* sp. 2 were shared between sympatric Baltic populations of gray and ringed seals.

**FIGURE 4:**
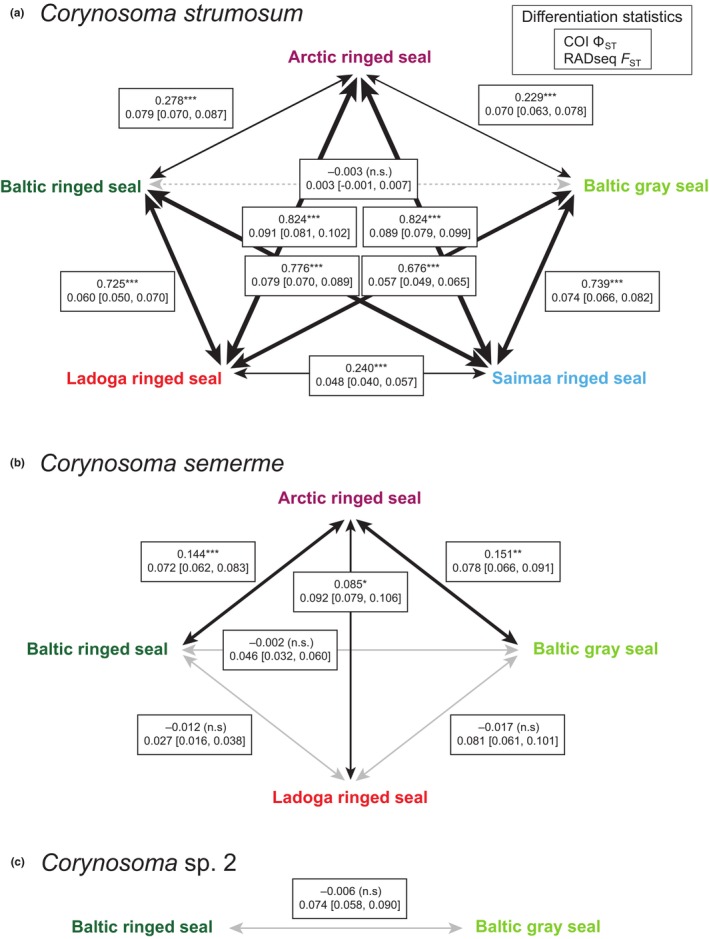
Pairwise estimates of genetic differentiation between population samples collected from different seal host (sub)species within (a) *C. strumosum*, (b) *C. semerme*, and (c) *Corynosoma* sp. 2. Estimates represent Φ_ST_ for mitochondrial COI barcode sequences and *F*_ST_ for RADseq SNPs (see legend). Asterisks next to Φ_ST_ values denote statistical significance (***p < .001, **p < .01, *p < .05, n.s. = not significantly different from zero). Numbers in square brackets after *F*_ST_ values represent the lower and upper limits of the 95% confidence intervals of each estimate. Thicknesses of black lines in (a) and (b) are proportional (within species) to statistically significantly non‐zero mtDNA Φ_ST_ values. Continuous gray lines represent differentiation that is statistically significantly non‐zero only for SNP markers, hatched gray lines denote differentiation that is statistically non‐significant for both COI and SNP markers.

Analyses of COI barcode variation revealed weak but statistically significant population‐genetic differentiation within two of the three *Corynosoma* species that were found in multiple hosts (Figure 4). Overall differentiation across hosts and geographical areas was particularly clear within *C. strumosum* (overall Φ_ST_ = 0.683, *p* < .0001). Pairwise estimates of differentiation were highest between the two landlocked populations and their marine relatives, while the populations from lakes Saimaa and Ladoga were less differentiated from each other (Figure 4a). *Corynosoma strumosum* population samples collected from Baltic ringed and gray seals did not differ from each other, but both were genetically differentiated from the population collected from Arctic ringed seal (Figure 4a). Population‐genetic structuring was substantially weaker but still statistically significant within *C. semerme* (overall Φ_ST_ = 0.035, *p* < .0001). However, this result mainly reflects the differentiation of *C. semerme* samples originating from Arctic ringed seals from the populations in Baltic ringed and gray seals and Ladoga ringed seals, while Φ_ST_ values estimated across the latter three populations were not statistically significantly different from zero (Figure 4b). Within *Corynosoma* sp. 2, population samples collected from Baltic ringed and gray seals did not differ from each other (Figure 4c). The *Corynosoma* sp. 2 population occurring in Arctic ringed seals was represented by a single individual that was excluded from the statistical analysis, but we note that the individual was placed as sister to the samples from Baltic ringed and gray seals in the NJ tree (Appendix S3: Figure S3.1A).

The ML tree based on concatenated RADseq data showed clear grouping of individuals by seal host population within all three *Corynosoma* species found in more than one seal host (sub)species (Figure 3b). *Corynosoma strumosum* individuals that originated from lakes Saimaa and Ladoga grouped according to lake and then together, collectively forming a sister group to individuals from Baltic gray and Baltic ringed seals. Specimens from Arctic ringed seals formed a grade with respect to the Baltic and freshwater specimens. The single individual of *Corynosoma* sp. 2 found from an Arctic ringed seal was placed as sister to the specimens originating from Baltic gray and Baltic ringed seals. Within *C. semerme*, individuals from Arctic ringed seals grouped together as sister to a group formed by partially intermixed individuals from Baltic gray seals and Baltic and Ladoga ringed seals.

Based on the species‐level RADseq datasets (Appendix S4: Figure S4.3), overall population differentiation within *C. strumosum* was estimated at *F*_ST_ = 0.067, and the bootstrapped 95% confidence interval of the estimate did not overlap with zero (95% CI = 0.061–0.072). When considering pairwise *F*_ST_ values across host (sub)species, differentiation between *C. strumosum* collected from Baltic ringed and gray seals was low and did not differ from zero statistically (*F*_ST_ = 0.003 [−0.001–0.007]), but all other values were statistically different from zero (Figure 4a). The greatest pairwise genetic differences were found between the population from Arctic ringed seals and those from lakes Ladoga and Saimaa, while the populations in the two lakes were relatively weakly differentiated from each other. Between‐population *F*_ST_ estimates were statistically significantly correlated with Φ_ST_ estimates calculated from the COI barcode data (*r* = .710, *p* = .019). The SNP‐based Admixture analysis indicated three ancestral populations within *C. strumosum*: from the Arctic, Baltic Sea, and Lake Saimaa (Appendix S4: Figure S4.5). The population of Lake Ladoga appeared admixed by both Baltic and Lake Saimaa ancestry and, in general, each population contained highly admixed individuals.

Overall population differentiation was statistically significant also for *C. semerme* (*F*_ST_ = 0.066 [0.058–0.075]). For this species, the lowest pairwise value occurred between the samples from Baltic and Ladoga ringed seals, but the 95% confidence interval of the estimate overlapped with that of the differentiation across Baltic ringed and Baltic gray seals (Figure 4b). Differentiation was higher between populations from Arctic and Ladoga ringed seals than between those from Arctic ringed seal and the two Baltic seal species, but again, the 95% confidence intervals of the pairwise *F*_ST_ estimates overlapped with each other. Between‐population estimates of SNP and COI differentiation within *C. semerme* were not statistically significantly correlated (*r* = .534, *p* = .326). For *Corynosoma* sp. 2, a meaningful test of differentiation could be done only between the population samples from Baltic ringed and Baltic gray seals; differentiation was statistically significant in this case (Figure 4c).

### 3.3 Genetic diversity

The studied *Corynosoma* species differed in their intraspecific mitochondrial diversity. The mean pairwise genetic distance of COI barcode sequences within *C. strumosum* was from 3 to 10.5 times higher than in the other species (Appendix S3: Table S3.2). This was reflected in the TCS haplotype network, in which—contrary to the high variation found within *C. strumosum—*sequences belonging to *C. semerme* and *Corynosoma* sp. 2 formed well‐separated haplotype groups with simple star‐like shapes (Figure 3a, Appendix S3: Figure S3.2). In the three *Corynosoma* species that were found in multiple seal (sub)species, estimates of within‐population gene and nucleotide diversity were in general similar when considering the relatively wide standard deviations of the values (Table 1). However, within *C. strumosum*, nucleotide diversity in the landlocked Saimaa and Ladoga populations was lower than in the three marine populations (Table 1).

**TABLE 1:** Sample sizes and mitochondrial and SNP‐based diversity indices for population samples of four *Corynosoma* species in four ringed seal subspecies and Baltic gray seals.

*Corynosoma* species, marker, *N*, and diversity indices	Host (sub)species				
	Saimaa ringed seal	Ladoga ringed seal	Baltic ringed seal	Arctic ringed seal	Baltic gray seal
*C. strumosum*					
mtDNA					
*N*	176	39	67	26	82
*N* haplotypes	39	23	53	17	62
Gene diversity	0.905 (0.012)	0.888 (0.046)	0.985 (0.007)	0.960 (0.021)	0.981 (0.008)
Nucleotide diversity	0.004 (0.003)	0.004 (0.003)	0.012 (0.006)	0.007 (0.004)	0.014 (0.007)
SNPs					
*N*	12	11	14	14	12
*H*_e_	0.075	0.073	0.076	0.094	0.085
*C. semerme*					
mtDNA					
*N*	–	22	38	11	75
*N* haplotypes	–	12	18	5	25
Gene diversity	–	0.762 (0.099)	0.728 (0.081)	0.764 (0.107)	0.609 (0.068)
Nucleotide diversity	–	0.002 (0.002)	0.001 (0.001)	0.004 (0.003)	0.002 (0.001)
SNPs					
*N*	–	4	4	4	4
*H*_e_	–	0.137	0.157	0.195	0.163
*Corynosoma* sp. 2					
mtDNA					
*N*	–	–	29	1	8
*N* haplotypes	–	–	14	1	5
Gene diversity	–	–	0.704 (0.097)	–	0.786 (0.151)
Nucleotide diversity	–	–	0.002 (0.001)	–	0.003 (0.002)
SNPs					
*N*	–	–	4	1	4
*H*_e_	–	–	0.076	–	0.085
*Corynosoma* sp. 1					
mtDNA					
*N*	–	–	–	4	–
*N* haplotypes	–	–	–	3	–
Gene diversity	–	–	–	0.833 (0.222)	–
Nucleotide diversity	–	–	–	0.007 (0.005)	–
SNPs					
*N*	–	–	–	3	–
*H*_e_	–	–	–	0.363	–

*Note*: Numbers in parentheses show standard deviations for estimates of mtDNA gene and nucleotide diversity.

In line with the mitochondrial results, the fineRADstructure analysis estimated lower co‐ancestry (and hence higher genotypic variation) for *C. strumosum* than for the three other species (Figure 2). Based on the SNP data, mean expected heterozygosity of *C. strumosum* was lowest in the two lake populations, but the estimates were not statistically significantly different from each other or from that of the population sample originating from Baltic ringed seals (Table 1). However, expected heterozygosity was statistically significantly higher in the population from the Baltic gray seal than from these three populations, and the diversity of the Arctic population was higher than in the other four populations (all *p* < .0013 after sequential Bonferroni correction). Within *C. semerme*, expected heterozygosity was lowest in the Ladoga population and highest in the Arctic, and the estimates of all populations were statistically significantly different from each other (Table 1; all pairwise *p* < .037 after sequential Bonferroni correction). Expected heterozygosity was likewise statistically significantly different in the populations of *Corynosoma* sp. 2 originating from Baltic ringed and Baltic gray seals, although the absolute values of the estimates were relatively similar (Table 1; *p* < .0001).

## 4 DISCUSSION

Parasite species richness as well as genetic diversity within parasite species are expected to be influenced by the phylogeographical histories and population sizes of their hosts (Blakeslee et al., 2020; Loiseau et al., 2017; Pérez‐Rodríguez et al., 2013). In the present study, the sequential colonization pattern and widely differing sizes of the focal northern European seal populations allowed us to trace how the species richness and intraspecific genetic composition of acanthocephalan parasites have changed during postglacial geographical and ecological shifts of their seal hosts. First, using a combination of DNA barcoding and RADseq genotyping, we determined how many *Corynosoma* species exist in the study system and within each seal population. According to our Hypothesis (i), we expected to find a gradient of decreasing species diversity from the abundant Arctic ringed seal to the small and isolated seal populations of lakes Saimaa and Ladoga. Next, we took a closer look at geographical and host‐associated differentiation as well as population‐level genetic diversity within those *Corynosoma* species that were found in multiple seal (sub)species. In this case, we expected to find geographical and/or host‐associated population‐genetic structuring within widespread *Corynosoma* species (Hypothesis (ii)), as well as a gradient of genetic diversity from the Arctic toward the small landlocked populations (Hypothesis (iii)). Below, we relate our findings to results from prior genetic studies of northern European ringed seals as well as to diversity patterns observed in other differentially isolated host–parasite systems.

### 4.1 *Corynosoma* species delimitation

Inference of parasite community richness and intraspecific genetic variation requires reliable delimitation of species (Stefan et al., 2018). In the case of *Corynosoma* and other acanthocephalans, many authors have pointed out the difficulties in identifying species, mainly due to their small size, reduced morphology, and the confusing intraspecific variability of morphological characters (Hernández‐Orts et al., 2022; Leidenberger et al., 2019; Li et al., 2019). Not surprisingly, studies based on DNA barcoding and targeted sequencing of the nuclear ribosomal ITS region have increasingly revealed the presence of cryptic species within *Corynosoma* (Waindok et al., 2018) and other acanthocephalan parasites (Rojas‐Sánchez et al., 2023; Steinauer et al., 2007; Zittel et al., 2018). While the small size of *Corynosoma* species has thus far limited the use of genome‐level markers, our results show that combining COI barcoding with genotypic data obtained using the novel 3RAD protocol of Bayona‐Vásquez et al. (2019) allows robust species delimitation and avoids the weaknesses of single‐gene approaches (Cháves‐González et al., 2022; Nadler & León, 2011).

Our species delimitation analyses, based on the most comprehensive sampling of *Corynosoma* specimens and genetic data to date, show that *C. magdaleni* does not exist in northern Europe. Instead, the patterns of genetic variation in the mtDNA and RADseq datasets evidently reflect spatial population divergence within the Holarctic *C. strumosum*. *Corynosoma magdaleni* was originally described from Canada in 1958 (Montreuil, 1958), but was from the 1980s onwards reported from the Baltic Sea (Delyamure et al., 1980; Leidenberger et al., 2019; Nickol et al., 2002; Valtonen et al., 2004) and lakes Saimaa and Ladoga (Sinisalo et al., 2003; Valtonen et al., 2004), and was believed to have been misidentified as *C. strumosum* in earlier studies from the region (e.g., Delyamure et al., 1980; Valtonen & Helle, 1988). In our results, the slight divergence between the two main mitochondrial barcode clusters within *C. strumosum* (Figure 3a, Appendix S3: Figure S3.1A) is not reflected in multilocus RADseq genotypes (Figures 2 and 3b). It is also noteworthy that *C. “magdaleni”* and *C. strumosum* individuals could not be consistently separated in the morphological blind test (Appendix S2) and that the two putative groups had similar distributions within seal intestines (Appendix S2: Figure S2.1). Molecular analysis of *Corynosoma* communities from the type locality of *C. magdaleni* in the Northwest Atlantic may eventually allow recognition of *C. magdaleni* as a junior synonym of *C. strumosum*.

Apart from *C. strumosum* and *C. semerme*, we found two divergent genetic groups—*Corynosoma* sp. 1 and 2—that have no counterparts in GenBank. Direct concordance between COI and RADseq genetic variation indicates that these groups constitute distinct species (Figures 2 and 3, Appendix S3: Figure S3.1). The morphological distinctness of *Corynosoma* sp. 1 remains to be investigated, as all four individuals belonging to this species originated from Arctic ringed seals and were, therefore, not included in the set of specimens sampled for our morphological blind test (Appendix S2). A detailed morphological study of *Corynosoma* sp. 2 was likewise not possible, but the results of the blind test suggest that *Corynosoma* sp. 2 is morphologically indistinguishable from *C. strumosum*. Notably, *Corynosoma* sp. 2 individuals were most often found in the posterior parts of the small intestine, indicating intra‐host niche segregation among *C. strumosum*, *C. semerme*, and *Corynosoma* sp. 2 (Appendix S2).

The barcode sequences of *Corynosoma* sp. 1 and *Corynosoma* sp. 2 did not correspond to the sequences of “Candidatus *Corynosoma nortmeri*” deposited by Waindok et al. (2018) from the North Sea population of harbor seals (Appendix S3: Figure S3.2), but they could represent some of the *Corynosoma* species that have been listed from seals in the Arctic (see Kelly et al., 2010; Kuzmina et al., 2012; Stryukov, 2000) that still lack public genetic data. Obtaining a full understanding of *Corynosoma* diversity in northern Europe will—in addition to the use of efficient genome‐level markers—require expanding sampling to encompass all main geographic areas and seal host species. In this respect, the orthologous RAD loci discovered in the present study can be used for designing capture baits for the RADcap method (Hoffberg et al., 2016) or amplification primers for GT‐seq panels (Bootsma et al., 2020; Campbell et al., 2015), both of which enable large‐scale genotyping at low cost.

### 4.2 Determinants of *Corynosoma* species richness

Seal populations inhabiting brackish and freshwater habitats are expected to harbor species‐poor parasite communities for several reasons. First, in accordance with a broad interpretation of the theory of island biogeography (Dallas & Jordano, 2021; Poulin, 2014), large populations of oceanic seals should have a richer parasite fauna because they occupy a larger area and are thus more likely to encounter and be colonized by parasites from other host species. Secondly, particular species of parasites may be excluded from new environments because of unfavorable conditions for either the parasites or some of their intermediate hosts (Hopper et al., 2014; Poulin, 1997; Torchin & Lafferty, 2009). This possibility is particularly relevant for seal‐infecting intestinal parasites that have complex life cycles involving relatively fixed sequences of intermediate and paratenic hosts, as at least some of the hosts from each step of the life cycle have to be able to survive in the novel environments. Additionally, the time since the establishment of these seal populations may not have been sufficient for acquiring local parasite species (cf. Gendron et al., 2012; Telfer & Bown, 2012). Finally, as freshwater seal populations are usually small and isolated, the abundance of seals or one or more intermediate hosts can easily drop below thresholds necessary for maintaining populations of certain parasite species (cf. Lloyd‐Smith et al., 2005; Romeo et al., 2021; Torchin et al., 2003; Zelmer, 2014).

Local *Corynosoma* community richness was in general agreement with the expectations of our Hypothesis (i), which was based on the colonization sequence and the current population sizes of the focal seal (sub)species (Figure 1c). Thus, our results confirmed the presence of four species of *Corynosoma* parasites in Arctic ringed seals and three in Baltic ringed and gray seals. In comparison, we found only two species in Ladoga ringed seals and a single species in Saimaa ringed seals (Figure 3). We note that the gradient in parasite diversity is, in fact, likely to be steeper than observed here, as observed parasite community richness is generally influenced by sample size (Teitelbaum et al., 2020; Walther et al., 1995), and the sample size from the Arctic was lower than for the other populations (Table 1). In their status review of ringed seal subspecies, Kelly et al. (2010) listed nine acanthocephalan species from Arctic ringed seals, but elucidating the true species count will require further genetic studies.

Available evidence suggests that other species‐rich groups of ringed seal parasites exhibit parallel gradients in community diversity: many nematodes, trematodes, and cestodes found in seals in the Arctic are rare or have not been recorded in ringed seals inhabiting the Baltic Sea or lakes Saimaa and Ladoga (Delyamure et al., 1980; Kunnasranta et al., 2021; Nyman et al., 2021; Sinisalo et al., 2003). For cestodes, the low species richness in landlocked seals appears to follow from life‐cycle disruption caused by lack of intermediate or paratenic hosts (Nyman et al., 2021), and this might also be the case for nematodes. For example, nematodes belonging to the *Contracaecum osculatum* species complex that infect Arctic and Baltic seals predominantly circulate through pelagic or semi‐pelagic cod species (Gadidae) and clupeids (Clupeidae) (Johansen et al., 2010; Zuo et al., 2016, 2018), which are absent from freshwater habitats in northern Europe.

By contrast, the *Corynosoma* diversity gradient revealed by our study does not seem to be explained by simple life‐cycle disruption. The postglacial survival of *C. strumosum* in lakes Saimaa and Ladoga appears to have been enabled by the presence of *Monoporeia affinis* and several other glacial relict amphipods in both lakes (Särkkä et al., 1990), as well as by the wide range of marine and freshwater fish species that can act as paratenic hosts for *C. strumosum* (Anikieva et al., 2018 and reference therein). The same intermediate and paratenic hosts are also suitable for *C. semerme* (Leidenberger et al., 2020), which is nevertheless absent from Lake Saimaa. Hence, the loss of *C. semerme* from Lake Saimaa may simply be a result of stochastic effects during colonization (as suggested by Sinisalo et al., 2003) or past fluctuations in the size of the Saimaa ringed seal population (cf. Nyman et al., 2014). Elucidating whether the distributions of *Corynosoma* sp. 1 and 2 are limited by environmental factors (in particular, salinity), life‐cycle disruption, or host population size will require molecular screening of *Corynosoma* communities in intermediate and paratenic hosts in the Arctic and in the Baltic Sea.

### 4.3 Host and *Corynosoma* population‐genetic structure

Host movement is considered to be a major determinant of parasite gene flow (Froeschke & von der Heyden, 2014; García‐Varela et al., 2021; Nadler & León, 2011). However, the concordance between the population‐genetic structures of parasites and definitive hosts may be disrupted by parasite dispersal via highly mobile intermediate or alternative hosts (Huyse et al., 2005; Jones & Britten, 2010; Mazé‐Guilmo et al., 2016; Witsenburg et al., 2015). Fitting our Hypothesis (ii), we found statistically significant population‐genetic structuring in mitochondrial COI sequences and/or RADseq SNP variation within all three *Corynosoma* species that were found in more than one seal (sub)species. Reflecting geographical isolation, the Arctic population was divergent from the Baltic Sea and freshwater populations of both *C. strumosum* and *C. semerme* (Figure 4a,b). Within *Corynosoma* sp. 2, the placement of the single Arctic specimen as sister to the Baltic Sea individuals in the phylogenetic trees estimated based on both mtDNA (Appendix S3: Figure S3.1) and RADseq (Figure 3b) data suggests that large‐scale geographical structuring follows the same pattern within this species. Similar to their seal hosts (Heino et al., 2023; Löytynoja et al., 2023; Palo et al., 2001; Peart et al., 2020), gene flow between the Arctic and Baltic populations of *Corynosoma* parasites, therefore, seems to be too low to prevent intraspecific genetic differentiation. The dependence of ringed seals on sea ice for constructing subnivean resting and breeding lairs during the winter makes a large portion of the ice‐free Norwegian coastline, as well as the southern parts of the Baltic Sea, unsuitable for permanent habitation by ringed seals (Figure 1c) (Oksanen et al., 2015). Despite the resultant wide gap in ringed seal distribution, some gene flow in *Corynosoma* parasites presumably still occurs through a series of different paratenic and final hosts. For example, although Baltic gray and harbor seals rarely migrate beyond the Baltic Sea (Dietz et al., 2013; Oksanen et al., 2014), their distributions in the southwestern part of the basin overlap with North Sea populations (Fietz et al., 2016), which may facilitate parasite dispersal.

In all three *Corynosoma* species present in the Baltic Sea, mtDNA differentiation was absent between populations collected from gray and ringed seals. These two seal host species differ in their dietary preferences (Scharff‐Olsen et al., 2019), but at least in the case of *C. strumosum* and *C. semerme*, the wide range of paratenic hosts (Anikieva et al., 2018; Leidenberger et al., 2020; Sinisalo et al., 2003; Valtonen et al., 2001) could promote gene flow at a level high enough to homogenize the entire Baltic population. Nevertheless, RADseq SNPs exhibited weak but statistically significant host‐associated differentiation within Baltic *C. semerme* and *Corynosoma* sp. 2 (Figure 4). More detailed sampling would be required to confirm the reasons underlying these signatures, but they could conceivably reflect a combination of intraspecific spatial differentiation and differences in the sampling locations of Baltic ringed and gray seals in the dataset.

The genetic similarity of the *C. strumosum* populations in lakes Saimaa and Ladoga (Figures 3 and 4) is surprising because it contrasts with the postglacial emergence history of the lakes. Lake Saimaa was formed and presumably colonized by ringed seals thousands of years before the separation of Lake Ladoga from the Baltic Sea basin, meaning that the divergence of the Saimaa population should precede the split of the Ladoga and Baltic Sea populations (Löytynoja et al., 2023; Saarnisto, 2011; Ukkonen et al., 2014). The two lakes are connected by the c. 150 km long Vuoksi River, which, even before the construction of a series of hydroelectric dams from the 1920s onwards (Jormola et al., 2016), was impassable for seals due to its many steep rapids. Gene flow between the *C. strumosum* populations of the two lakes could, however, conceivably occur through dispersal within paratenic fish hosts. Our finding of “lake haplotypes” in the Baltic Sea but not vice versa (Figure 3a) suggests that downstream gene flow takes place from Lake Ladoga to the Baltic Sea through the *c*. 70 km long Neva River. It should be noted, however, that differentiation statistics (Figure 4a) and the admixture analysis (Appendix S4: Figure S4.5) suggest a higher proportion of Baltic ancestry within Lake Ladoga than within Saimaa. These latter patterns resemble results from genetic analyses of the seal hosts (Löytynoja et al., 2023; Nyman et al., 2014). The possibility of upstream gene flow from the Baltic Sea to Lake Ladoga is supported by our finding that the *C. semerme* population of the lake is likewise weakly differentiated from the one infesting Baltic ringed seals (Figure 4b).

In the TCS network based on COI barcode sequences, the *C*. “*magdaleni*” clade formed by all C. *strumosum* individuals from lakes Ladoga and Saimaa, as well as six individuals from the Baltic Sea, was separated from the Baltic and Arctic haplotypes by more than 11 mutational steps (Figure 3a). These two clades are, therefore, genetically more distant than are North and South American barcode sequences of the widespread generalist *C. australe* (García‐Varela et al., 2021). Notably, when placed in a broader geographical context, *C. strumosum* mtDNA haplotypes representing the Lake Ladoga + Lake Saimaa clade were more closely related to Pacific and Arctic haplotypes than to the main group of Baltic haplotypes (Appendix S3: Figure S3.2). This intriguing finding suggests that the two lake‐endemic *C. strumosum* populations retain ancestral genetic variation that in the Baltic Sea has been erased by younger haplotypes that might have arrived when gray seals recolonized the Baltic Sea during the Bronze or Iron Ages after being hunted to local extinction by humans during the Mesolithic (Ahlgren et al., 2022). While similar replacement of the Baltic ringed seal population is not apparent in archeological data (Ukkonen et al., 2014), recent genetic studies have increasingly converged toward the conclusion that the genetic composition of the extant Baltic ringed seal population differs from the one that was present during colonization of Lake Saimaa (Heino et al., 2023; Löytynoja et al., 2023). As pointed out by Whiteman and Parker (2005), genetic analyses of host‐specific parasites provide a potentially powerful tool for illuminating both deep evolutionary histories and recent population subdivisions of their hosts (*see also* Gagne et al., 2022; Koop et al., 2014; Nieberding & Olivieri, 2007; Whiteman et al., 2007). Evidently, community‐level comparative genetic analyses of *Corynosoma* and other specialist seal parasites could provide important insights into the colonization history of seals in northern Europe.

### 4.4 Host and *Corynosoma* genetic diversity

Sequential colonization events (Clegg et al., 2002; Pruett & Winker, 2005), population isolation (Kardos et al., 2021; Lehnen et al., 2021), and recent anthropogenic bottlenecks (Dussex et al., 2018; Spielman et al., 2004; von Seth et al., 2021) can erode genetic diversity within animal populations. In our focal seals, such effects have led to a gradient of genetic variation from the highly diverse Arctic ringed seal to the genetically very uniform Saimaa ringed seal (Löytynoja et al., 2023; Nyman et al., 2014; Peart et al., 2020; Stoffel et al., 2018). Fitting the predictions of our Hypothesis (iii), we found that the Baltic and lake populations of *C. strumosum* are significantly less diverse than the Arctic population and that the lowest estimates of expected SNP heterozygosity and mtDNA nucleotide diversity within the species are found in the two landlocked populations (Table 1). The reduced genetic diversity of the freshwater populations is especially evident in COI barcode variation (Figure 3a, Table 1), which is expected given that the effective gene number of mtDNA is one‐fourth of that of nuclear DNA (Nadler & León, 2011). The loss of genetic diversity is less pronounced within *C. semerme* but expected heterozygosity in SNP markers was also highest in the Arctic and lowest in the Ladoga population in this species (Table 1).

Genetic surveys have shown that the Saimaa ringed seal population has lost 44–69% of its nuclear heterozygosity (Löytynoja et al., 2023; Palo et al., 2003) and 47%–89% of its mtDNA nucleotide diversity (Heino et al., 2023; Kunnasranta et al., 2021; Valtonen et al., 2012) in comparison with the Baltic source population. However, especially within *C. strumosum*, the declines are much weaker than in the seal hosts. A likely explanation is that bottleneck effects and long‐term genetic drift have been stronger in the seals than in *Corynosoma*, which presumably can have large local effective population sizes due to high infestation intensities (Sinisalo et al., 2003; Valtonen et al., 2004) and effective intra‐host population mixing due to their complex life cycles (Figure 1d). High infrapopulation sizes and efficient outbreeding resulting from life cycles involving multiple intermediate hosts have been implicated as factors underlying high *N*_e_ in, for example, trichostrongylid nematodes (Blouin et al., 1995; Huyse et al., 2005) and *Diplostomum* trematodes (Rauch et al., 2005). In general, the complex life history and effective outbreeding of acanthocephalans and many other intestinal parasite taxa are expected to lead to population‐genetic patterns that are fundamentally different from those of their hosts as well as directly transmitted ectoparasites (Criscione & Blouin, 2005; Janecka et al., 2021). Specialist ectoparasites such as lice may form genetically distinct infrapopulations on single host individuals (Koop et al., 2014; Virrueta Herrera et al., 2022), and species‐level effective population sizes may be correlated with host body size due to more severe inbreeding on small‐bodied hosts (Doña & Johnson, 2023).

## 5 CONCLUSIONS

Studies of host–parasite systems that have experienced distributional shifts, geographical range fragmentation, and population declines in the past can help us predict how host and parasite community richness and genetic diversity will be influenced by anthropogenic environmental change in the future (Carlson et al., 2020; Cook et al., 2017; Velo‐Antón et al., 2012). In northern Europe, *Corynosoma* community richness is explained by the postglacial colonization history and population sizes of their seal hosts, although historical contingencies and availability of intermediate and additional final hosts most likely also play a role. At the intraspecific level, *Corynosoma* populations inhabiting different geographical areas and seal (sub)species are genetically differentiated. While population differentiation shows broad concordance with spatial patterns found in the seal hosts, some discordances are also evident. In particular, the loss of genetic diversity in landlocked *Corynosoma* populations appears to have been less drastic than in seals. Retention of genetic variation in *Corynosoma* may have been facilitated by high within‐host population sizes and efficient outbreeding arising from continuous mixing of parasite infrapopulations through intermediate hosts. On long‐time scales, such differential erosion of genetic variation in fragmented host–parasite systems could fundamentally alter adaptive potential in coevolving hosts and parasites (Papkou et al., 2021, 2016; White et al., 2021). In the case of endangered host–parasite systems, differential loss of genetic variation can also have more immediate conservation implications, if local parasite abundance is linked to genetic diversity of the parasites (Benesh, 2019; Forsman, 2014; Fredericksen et al., 2021; Johnson & Hoverman, 2012) as well as their hosts (Ekroth et al., 2019; Gibson & Nguyen, 2021; Hoffman et al., 2014; Whitehorn et al., 2010).

This study demonstrates the power of the 3RAD genotyping method of Bayona‐Vásquez et al. (2019) for delimiting species and studying the population‐genetic structures of small parasites that are challenging for most other genome‐level methods. However, while 3RAD genotyping is applicable to most organismal groups with little taxon‐specific modification, elucidating the processes that shape global and local diversity of species‐rich marine parasite communities will require integration of morphological, genetic, and ecological information. Additionally, and perhaps most importantly, very broad geographical sampling spanning many host lineages will be needed in order to adequately cover the full spectrum of putative species (García‐Varela et al., 2021). Despite the practical challenges, filling this gap is necessary given the importance of including parasites in our effort to understand and combat global biodiversity loss (Carlson et al., 2020, 2017; Dunn et al., 2009; Dupouy‐Camet, 2016).

## AUTHOR CONTRIBUTIONS

**Ludmila Sromek:** Conceptualization (equal); data curation (lead); formal analysis (lead); funding acquisition (equal); investigation (equal); methodology (equal); project administration (supporting); resources (supporting); visualization (lead); writing – original draft (lead); writing – review and editing (supporting). **Eeva Ylinen:** Data curation (supporting); funding acquisition (equal); investigation (supporting); methodology (supporting); resources (lead); writing – review and editing (supporting). **Mervi Kunnasranta:** Conceptualization (supporting); funding acquisition (lead); investigation (supporting); project administration (equal); resources (equal); supervision (equal); writing – review and editing (supporting). **Simo N. Maduna:** Formal analysis (supporting); methodology (supporting); resources (supporting); writing – review and editing (supporting). **Tuula Sinisalo:** Investigation (supporting); resources (supporting); validation (supporting). **Craig T. Michell:** Investigation (supporting); methodology (supporting); resources (supporting). **Kit M. Kovacs:** Investigation (supporting); resources (supporting); writing – review and editing (supporting). **Christian Lydersen:** Investigation (supporting); resources (supporting). **Evgeny Ieshko:** Investigation (supporting); resources (supporting). **Elena Andrievskaya:** Investigation (supporting); resources (supporting). **Vyacheslav Alexeev:** Investigation (supporting); resources (supporting). **Sonja Leidenberger:** Investigation (supporting); methodology (supporting); writing – review and editing (supporting). **Snorre B. Hagen:** Resources (supporting); writing – review and editing (supporting). **Tommi Nyman:** Conceptualization (lead); data curation (supporting); formal analysis (supporting); funding acquisition (equal); investigation (equal); methodology (equal); project administration (lead); resources (equal); supervision (lead); visualization (supporting); writing – original draft (supporting); writing – review and editing (lead).

## CONFLICT OF INTEREST STATEMENT

The authors declare no conflicts of interest.

### OPEN RESEARCH BADGES

This article has earned an Open Data badge for making publicly available the digitally‐shareable data necessary to reproduce the reported results. The data is available at https://doi.org/10.5281/zenodo.7965559, GenBank accession numbers: OQ471326–OQ471903, NCBI Sequence Read Archive: BioProject PRJNA937090.

## Supporting information


Appendix S1

Appendix S2

Appendix S3

Appendix S4

## Data Availability

COI barcode sequences reported in this paper have been deposited in GenBank (accession numbers: OQ471326–OQ471903) and RADseq data in the NCBI Sequence Read Archive (SRA) under BioProject PRJNA937090. Alignments and data files used in the analyses are available on Zenodo (https://doi.org/10.5281/zenodo.7965559). The anterior halves of *Corynosoma* individuals analyzed in this study have been stored as vouchers in the Biobank of NIBIO Svanhovd.
