# Supplementary material for: Loss of species and genetic diversity during colonization: Insights from acanthocephalan parasites in northern European seals
Source: Ecol Evol. 2023 Oct 19;13(10):e10608. doi: 10.1002/ece3.10608 (PMC10585441; doi:10.1002/ece3.10608)
Supplement: Supplementary file 2 — Appendix S2 [file ECE3-13-e10608-s002.pdf]

Appendix S2

Morphological blind test

Pre-identification of specimens prior to molecular-genetic analyses can reveal whether morphological traits are reliable for determining the species identity of single individuals. Before our molecular analyses, a random set of 55 *Corynosoma* individuals collected from Saimaa and Baltic ringed seals and Baltic grey seals was pre-identified under a stereomicroscope by TS and then processed in a manner similar to all of the other samples.

Based on comparisons between the morphology-based identifications and assignments based on COI barcodes and RADseq genotyping results, all 19 *C. semerme* individuals were correctly identified to species (Table S2.1). Of the 36 remaining specimens, 14 were considered unidentifiable (juveniles, poorly preserved individuals, and specimens with a retracted proboscis), one was identified as *C. strumosum*, and 21 as *C. magdaleni*. Seven individuals identified as *C. magdaleni* (six from Lake Saimaa and one from the Baltic Sea) grouped within the *C. "magdaleni"* barcode cluster on the NJ tree (Appendix S3, Figure S3.1A). However, 14 Baltic specimens belonged either to the *Corynosoma* sp. 2 (eight individuals) or *C. strumosum s.str*. (six individuals) barcode clusters. Taken together, these results suggest that neither *C. strumosum s.str*. and *C. magdaleni* nor *C. strumosum* and *Corynosoma* sp. 2 can be distinguished consistently based on their morphology.

Distribution of *Corynosoma* species within seal intestines

For intestinal parasites, different parts of the gastrointestinal tract constitute a succession of microhabitats characterized by differential immune responses, gut microbiota, and availability of nutrients and dietary items at different stages of digestion (Shanebeck et al., 2020). The existence of intra-host spatial segregation is suggestive of niche specialization and can therefore constitute additional evidence of species distinctness. To explore this, we tested differences in the distribution of *Corynosoma* species within seal intestines using a randomization test. For this, we used 299 *Corynosoma* individuals collected from the Baltic ringed and grey seal populations, where three *Corynosoma* species were present and the intestinal section had been recorded. For the test, the locations of individual acanthocephalans were shuffled 10,000 times among gut sections (numbered from 1 to 13) that had samples within each seal individual, and differences in the medians of the location of each species pair were recorded after each randomization. Two-tailed *P* values were calculated by counting those differences that were equal to or more extreme than the absolute value of the observed difference in the medians.

The three *Corynosoma* species differed in their spatial distribution within the digestive tracts of Baltic ringed and grey seals (Figure S2.1). In the Baltic ringed seal, the median location of *C. semerme* differed from that of *C. strumosum* ($P < 0.0001$) and *Corynosoma* sp. 2 ($P = 0.0009$). The median location of *Corynosoma* sp. 2 likewise differed from that of *C. strumosum* ($P = 0.0089$). Within the digestive tracts of Baltic grey seals, the median locations of *C. strumosum* differed from that of *C. semerme* ($P < 0.0001$) and *Corynosoma* sp. 2 ($P = 0.0227$). In contrast, the median locations of *C. semerme* and *Corynosoma* sp. 2 did not differ ($P = 0.2005$), but this probably reflects low statistical power due to the small sample size of *Corynosoma* sp. 2 in the grey seal ($N = 8$).

The differing spatial distributions of *C. semerme* and *C. strumosum,* and the preference of *C. semerme* of the large intestine was expected based on previous studies (Nickol et al., 2002; Lakemeyer et al., 2020; Shanebeck et al., 2020). More importantly, the statistically significant difference between *C. strumosum* and *Corynosoma* sp. 2 identified in our study supports their distinctiveness and suggests subtle microhabitat segregation of also these *Corynosoma* species.

References

Lakemeyer, J., Lehnert, K., Woelfing, B., Pawliczka, I., Silts, M., Dähne, M., von Vietinghoff, V., Wohlsein, P., Siebert, U., 2020. Pathological findings in North Sea and Baltic grey seal and harbour seal intestines associated with acanthocephalan infections. Dis. Aquat. Organ. 138, 97–110. https://doi.org/10.3354/dao03440

Nickol, B.B., Helle, E., Valtonen, E.T., 2002. *Corynosoma magdaleni* in gray seals from the Gulf of Bothnia, with emended descriptions of *Corynosoma strumosum* and *Corynosoma magdaleni*. J. Parasitol. 88, 1222–1229. https://doi.org/10.2307/3285497

Shanebeck, K., Lakemeyer, J., Siebert, U., Lehnert, K., 2020. Habitat selection and populations of *Corynosoma* (Acanthocephala) in the intestines of sea otters (*Enhydra lutris*) and seals. J. Helminthol. 94, e211. https://doi.org/10.1017/S0022149X20000747

Table S2.1. Results of the morphological blind test.

Individual code	Species	Locality	Host (sub)species	COI cluster	Morphology blind test
**EY_374**	*C. strumosum*	Lake Saimaa	*P. h. saimensis*	*C. «magdaleni»*	*C. magdaleni*
**EY_375**	*C. strumosum*	Lake Saimaa	*P. h. saimensis*	*C. «magdaleni»*	Unidentifiable
**EY_376**	*C. strumosum*	Lake Saimaa	*P. h. saimensis*	*C. «magdaleni»*	*C. magdaleni*
**EY_377**	*C. strumosum*	Lake Saimaa	*P. h. saimensis*	*C. «magdaleni»*	*C. magdaleni*
**EY_378**	*C. strumosum*	Lake Saimaa	*P. h. saimensis*	*C. «magdaleni»*	Unidentifiable
**EY_379**	*C. strumosum*	Lake Saimaa	*P. h. saimensis*	*C. «magdaleni»*	*C. magdaleni*
**EY_380**	*C. strumosum*	Lake Saimaa	*P. h. saimensis*	*C. «magdaleni»*	Unidentifiable
**EY_381**	*C. strumosum*	Lake Saimaa	*P. h. saimensis*	*C. «magdaleni»*	*C. magdaleni*
**EY_382**	*C. strumosum*	Lake Saimaa	*P. h. saimensis*	*C. «magdaleni»*	Unidentifiable
**EY_383**	*C. strumosum*	Lake Saimaa	*P. h. saimensis*	*C. «magdaleni»*	*C. magdaleni*
**EY_384**	*C. strumosum*	Baltic Sea	*H. grypus*	*C. strumosum s.str.*	*C. magdaleni*
**EY_385**	*C. strumosum*	Baltic Sea	*H. grypus*	*C. strumosum s.str*	Unidentifiable juvenile
**EY_386**	*C. strumosum*	Baltic Sea	*H. grypus*	*C. «magdaleni»*	Unidentifiable
**EY_387**	*C. strumosum*	Baltic Sea	*H. grypus*	*C. strumosum s.str*	Unidentifiable
**EY_388**	*C. strumosum*	Baltic Sea	*H. grypus*	*C. strumosum s.str.*	Unidentifiable juvenile
**EY_389**	*C. strumosum*	Baltic Sea	*H. grypus*	*C. strumosum s.str.*	Unidentifiable juvenile
**EY_390**	*Corynosoma* sp. 2	Baltic Sea	*H. grypus*	*Corynosoma* sp. 2	*C. magdaleni*
**EY_391**	*C. strumosum*	Baltic Sea	*H. grypus*	*C. strumosum s.str.*	*C. magdaleni*
**EY_392**	*C. strumosum*	Baltic Sea	*H. grypus*	*C. strumosum s.str*	*C. magdaleni*
**EY_393**	*C. strumosum*	Baltic Sea	*H. grypus*	*C. «magdaleni»*	*C. magdaleni*
**EY_394**	*C. strumosum*	Baltic Sea	*P. h. botnica*	*C. strumosum s str*	Unidentifiable
**EY_395**	*C. strumosum*	Baltic Sea	*P. h. botnica*	*C. strumosum s str.*	Unidentifiable
**EY_396**	*C. strumosum*	Baltic Sea	*P. h. botnica*	*C. strumosum s str.*	Unidentifiable
**EY_397**	*C. strumosum*	Baltic Sea	*P. h. botnica*	*C. strumosum s str.*	*C. strumosum*
**EY_398**	*C. strumosum*	Baltic Sea	*P. h. botnica*	*C. strumosum s.str.*	Unidentifiable
**EY_399**	*C. strumosum*	Baltic Sea	*P. h. botnica*	*C. strumosum s.str.*	*C. magdaleni*
**EY_400**	*Corynosoma* sp. 2	Baltic Sea	*P. h. botnica*	*Corynosoma* sp. 2	*C. magdaleni*
**EY_401**	*C. strumosum*	Baltic Sea	*P. h. botnica*	*C. strumosum s str.*	*C. magdaleni*
**EY_402**	*C. strumosum*	Baltic Sea	*P. h. botnica*	*C. strumosum s.str.*	*C. magdaleni*
**EY_403**	*Corynosoma* sp. 2	Baltic Sea	*P. h. botnica*	*Corynosoma* sp. 2	*C. magdaleni*
**EY_404**	*Corynosoma* sp. 2	Baltic Sea	*P. h. botnica*	*Corynosoma* sp. 2	*C. magdaleni*
**EY_405**	*C. semerme*	Baltic Sea	*P. h. botnica*	*C. semerme*	*C. semerme*
**EY_406**	*Corynosoma* sp. 2	Baltic Sea	*P. h. botnica*	*Corynosoma* sp. 2	*C. magdaleni*
**EY_407**	*C. strumosum*	Baltic Sea	*P. h. botnica*	*C. strumosum s.str.*	Unidentifiable
**EY_408**	*C. semerme*	Baltic Sea	*P. h. botnica*	*C. semerme*	*C. semerme*
**EY_409**	*C. semerme*	Baltic Sea	*H. grypus*	*C. semerme*	*C. semerme*
**EY_410**	*C. semerme*	Baltic Sea	*H. grypus*	*C. semerme*	*C. semerme*
**EY_411**	*C. semerme*	Baltic Sea	*H. grypus*	*C. semerme*	*C. semerme*
**EY_412**	*C. semerme*	Baltic Sea	*H. grypus*	*C. semerme*	*C. semerme*
**EY_413**	*C. semerme*	Baltic Sea	*H. grypus*	*C. semerme*	*C. semerme*
**EY_414**	*Corynosoma* sp. 2	Baltic Sea	*H. grypus*	*Corynosoma* sp. 2	*C. magdaleni*
**EY_415**	*C. semerme*	Baltic Sea	*H. grypus*	*C. semerme*	*C. semerme*

**EY_416**	*C. semerme*	Baltic Sea	*H. grypus*	*C. semerme*	*C. semerme*
**EY_417**	*C. semerme*	Baltic Sea	*H. grypus*	*C. semerme*	*C. semerme*
**EY_418**	*C. semerme*	Baltic Sea	*H. grypus*	*C. semerme*	*C. semerme*
**EY_419**	*C. semerme*	Baltic Sea	*P. h. botnica*	*C. semerme*	*C. semerme*
**EY_420**	*C. semerme*	Baltic Sea	*P. h. botnica*	*C. semerme*	*C. semerme*
**EY_421**	*C. semerme*	Baltic Sea	*P. h. botnica*	*C. semerme*	*C. semerme*
**EY_422**	*C. semerme*	Baltic Sea	*P. h. botnica*	*C. semerme*	*C. semerme*
**EY_423**	*Corynosoma* sp. 2	Baltic Sea	*P. h. botnica*	*Corynosoma* sp. 2	*C. magdaleni*
**EY_424**	*Corynosoma* sp. 2	Baltic Sea	*P. h. botnica*	*Corynosoma* sp. 2	*C. magdaleni*
**EY_425**	*C. semerme*	Baltic Sea	*P. h. botnica*	*C. semerme*	*C. semerme*
**EY_426**	*C. semerme*	Baltic Sea	*P. h. botnica*	*C. semerme*	*C. semerme*
**EY_427**	*C. semerme*	Baltic Sea	*P. h. botnica*	*C. semerme*	*C. semerme*
**EY_428**	*C. semerme*	Baltic Sea	*P. h. botnica*	*C. semerme*	*C. semerme*

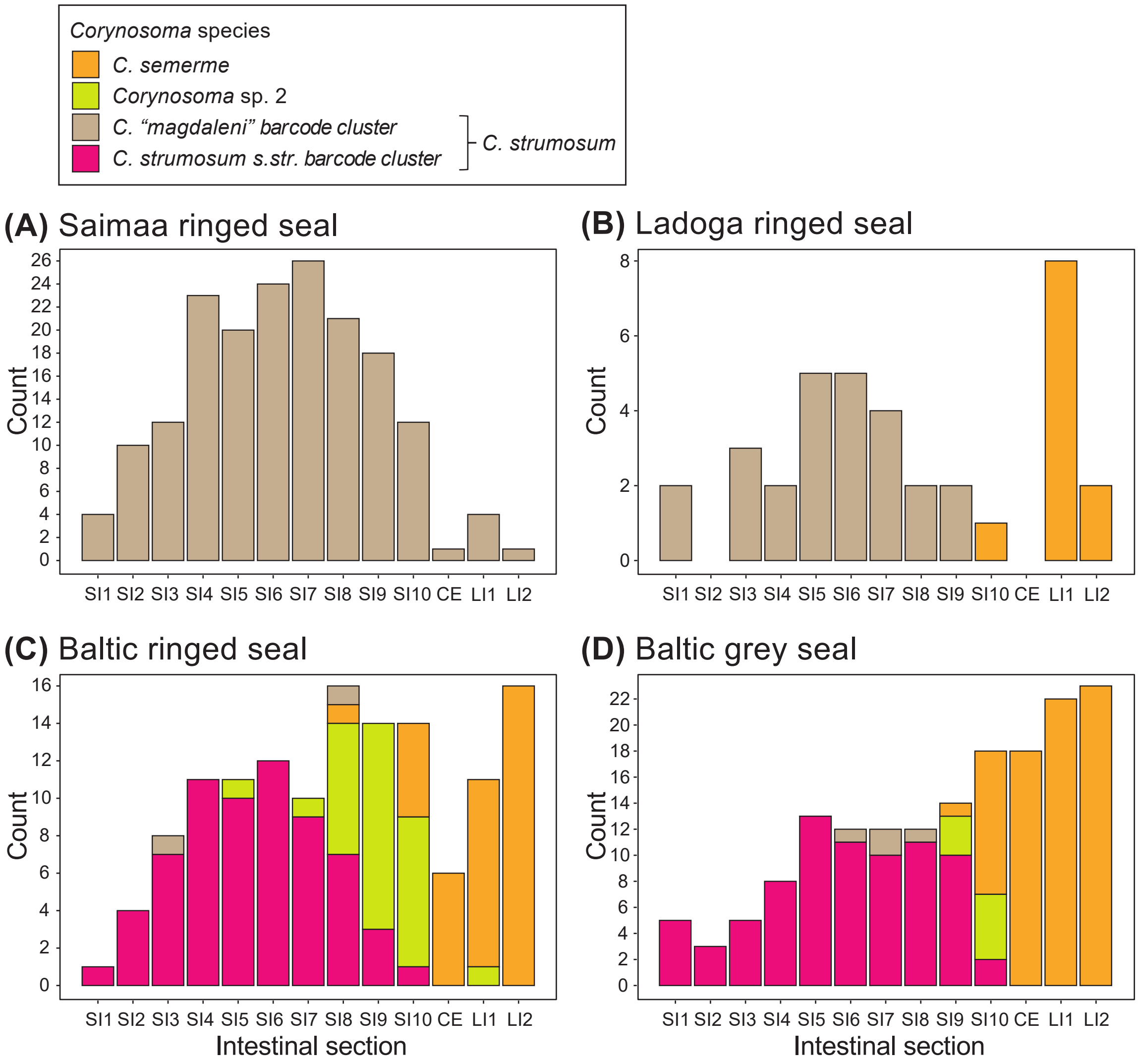

Figure S2.1. Occurrence of *Corynosoma* species in different parts of the intestines of three ringed seal subspecies (A–C) and Baltic grey seals (D). Codes below the X axis denote intestinal sections (SI – small intestine, CE – caecum, LI – large intestine). Note that although individuals belonging to the *C. strumosum s.str.* and *C. "magdaleni"* barcode clusters are here shown separately to illustrate their similar distributions, they were united under the final species assignment (*C. strumosum*) in the statistical tests.
