# Supplementary material for: Loss of species and genetic diversity during colonization: Insights from acanthocephalan parasites in northern European seals
Source: Ecol Evol. 2023 Oct 19;13(10):e10608. doi: 10.1002/ece3.10608 (PMC10585441; doi:10.1002/ece3.10608)
Supplement: Supplementary file 3 — Appendix S3 [file ECE3-13-e10608-s004.pdf]

Appendix S3

Sequencing of the mitochondrial COI barcode region

We amplified the 655-bp long standard DNA barcoding region of the mitochondrial COI gene using PCR primers T7P-LCO1490-GV (5'-**TAA TAC GAC TCA CTA TAG GG**A GTT CTA ATC ATA ARG ATA TYG G-3') and T3R-HCO2198 (5'-**ATT AAC CCT CAC TAA AG**T AAA CTT CAG GGT GAC CAA AAA ATC A-3') (Folmer et al., 1994; García-Varela et al., 2013). In addition to the universal binding site, these primers include T7P and T3R tails (in bold font) that act as binding sites for universal sequencing primers (but see below). PCR was conducted in 30-µl reactions including 15 µl REDTaq ReadyMix PCR Reaction Mix (Sigma-Aldrich), 12 µl H2O, 1 µl of 10 µM forward and reverse primers (final concentration 0.33 µM for each), and 1 µl of DNA extract. The PCR cycling conditions were as follows: denaturation for 5 min at 92 °C, followed by 35 cycles of 1 min denaturation at 92 °C, 1 min annealing at 48 °C, and 1 min extension at 72 °C; followed by a final 5 min extension at 72 °C. PCR products were checked on 1.5% agarose gels stained with ethidium bromide before purification with the QIAquick PCR Purification Kit (Qiagen) following the manufacturer's protocol.

Purified products were Sanger sequenced at Macrogen Europe and then assembled and edited in Sequencher v. 4.9 (GeneCodes Corporation). Due to occasional sequencing failures using the universal T7P and T3R sequencing primers, we instead performed sequencing in both directions using the internal primers iF-10 (5'-TAT GTT TTG GTY AGG YTG TGG A-3') and iR-586 (5'-AMC TAG TMC TAA AAT TAC GAT C-3'). If the sequencing in either direction failed with these primers, we used the internal primers iF-1 (5'-GGT TTT ATG TAT GTT TTG GT-3') and/or iR-541 (5'-AGC CAA RAC AGG AAT AGT TAA-3'). We note that, while most *Corynosoma* barcode sequences published in GenBank are incomplete at one or both ends, the use of our new internal primers allows sequencing of the entire standard 655-bp DNA barcode region.

The non-focal acanthocephalans originating from bearded seals, which were included as outgroups in the RADseq run, were not COI barcoded using Sanger sequencing; rather, we constructed barcode sequences for these specimens based on the RADseq data. For this, we first mapped their raw sequence reads against the COI reference of *C. strumosum* using Bowtie2 (Langmead and Salzberg, 2012), then constructed consensus sequences from the mapped reads using SAMtools (Li et al., 2009).

BLAST results for COI sequences

Based on the NJ tree and comparisons with sequences in the GenBank database, 578 COI sequences generated in this study could be divided into five groups (Figure S3.1A). The first group consisted of

sequences having the highest percent identity (98.6–100%) with a published sequence of *C. magdaleni* (GenBank acc. no. EF467872). This group included all specimens from Saimaa ringed seals (170 from the small intestine (SI), 1 from the cecum (CE), and 5 from the large intestine (LI), 39 individuals from Ladoga ringed seals (all from the SI), 2 individuals from Baltic ringed seals (both from the SI), and 4 individuals from Baltic grey seals (all from the SI). The second group consisted of sequences having the highest percent identity (97.6–99.8%) with various sequences published under the name *C. strumosum*, and included 65 specimens from Baltic ringed seals, 26 from the Arctic ringed seals, and 78 from Baltic grey seals (all from the SI). This latter group also included individual EY_393 from a Baltic grey seal, which grouped as sister to the *C. "magdaleni"* cluster on the NJ tree (Figure S3.1A). Sequences of *C. "magdaleni"* and *C. strumosum s.str.* formed reciprocally monophyletic groups only when this borderline individual was considered to belong to *C. "magdaleni"*. Furthermore, while sequences assigned to *C. strumosum s.str.* and *C. "magdaleni*" form distinct clusters in the TCS haplotype network (Figure S3.2), GenBank sequences of *C. strumosum* from Pacific pinnipeds and fish (García-Varela and Pérez-Ponce de León, 2008; Lisitsyna et al., 2019; Sasaki et al., 2019) essentially bridge the apparent gap between these two clusters (Figure S3.2).

Barcodes belonging to the next two groups did not match any sequences available in databases, so we refer to them as *Corynosoma* sp. 1 and sp. 2. *Corynosoma* sp. 1 consisted of four individuals from the SI of Arctic ringed seals, whose barcodes matched published sequences of *C. strumosum* with only 94.9–95.6% identity. *Corynosoma* sp. 2 consisted of barcodes of 1 specimen originating from an Arctic ringed seal (SI), 29 specimens from Baltic ringed seals (28 from the SI and 1 from the LI), and 8 specimens from Baltic grey seals (all from the SI). In this case, the top BLAST hits (sequence identities of 90.9–92.3%) were to *C. strumosum* (LC465357 and LC465390), Candidatus *Corynosoma nortmeri* (MF001278), and *C. magdaleni* isolate Pv1NS (MF078642). Lisitsyna et al. (2019) pointed out that the latter two sequences differ by only a single nucleotide and considered Candidatus *Corynosoma nortmeri* (MF001278) as a representative of *C. magdaleni*. However, all of our 221 *C. magdaleni* sequences resembled the *C. magdaleni* sequence EF467872 deposited by García-Varela and Pérez-Ponce de León (2008), but are very different from the *C. magdaleni* isolate Pv1NS sequence published by Waindok et al. (2018) (Figure S3.2). *Corynosoma magdaleni* isolate Pv1NS has probably been entered into GenBank under the wrong name, because its amino acid sequence does not correspond to the description and alignment shown in Waindok et al. (2018). Based on our results, *C. "magdaleni"* should be synonymized with *C. strumosum*, but the species identity of *C. nortmeri* requires further clarification.

The last group was formed by sequences that had the highest identity (99.1–100%) to various published sequences of *C. semerme*. This group included 38 specimens from Baltic ringed seals (6 from the SI, 6 from the CE, and 26 from the LI), 22 from Ladoga ringed seals (1 from the SI and 21

from the LI), 11 from Arctic ringed seals (all from the LI), and 75 from the Baltic grey seals (12 from the SI, 18 from the CE, and 45 from the LI).

The non-focal *Corynosoma* specimens collected from bearded seals that were used as an outgroup in phylogenetic analyses based on RADseq data were identified by blasting them against the GenBank database. These individuals apparently represent *C. villosum*, as fragmented COI sequences (215–590 bp) that we were able to assemble from the RADseq reads had 97–100% identities to published sequences of this species.

References

Folmer, O., Black, M., Hoeh, W., Lutz, R., Vrijenhoek, R., 1994. DNA primers for amplification of mitochondrial cytochrome c oxidase subunit I from diverse metazoan invertebrates. Mol. Mar. Biol. Biotechnol. 3, 294–299.

García-Varela, M., Pérez-Ponce de León, G., 2008. Validating the systematic position of *Profilicollis* Meyer, 1931 and *Hexaglandula* Petrochenko, 1950 (Acanthocephala: Polymorphidae) using cytochrome c oxidase (cox 1). J. Parasitol. 94, 212–217. https://doi.org/10.1645/GE-1257.1

García-Varela, M., Pérez-Ponce de León, G., Aznar, F.J., Nadler, S.A., 2013. Phylogenetic relationship among genera of Polymorphidae (Acanthocephala), inferred from nuclear and mitochondrial gene sequences. Mol. Phylogenet. Evol. 68, 176–184. https://doi.org/10.1016/j.ympev.2013.03.029

Langmead, B., Salzberg, S.L., 2012. Fast gapped-read alignment with Bowtie 2. Nat. Methods 9, 357–359. https://doi.org/10.1038/nmeth.1923

Li, H., Handsaker, B., Wysoker, A., Fennell, T., Ruan, J., Homer, N., Marth, G., Abecasis, G., Durbin, R., 1000 Genome Project Data Processing Subgroup, 2009. The Sequence Alignment/Map format and SAMtools. Bioinformatics 25, 2078–2079. https://doi.org/10.1093/bioinformatics/btp352

Lisitsyna, O.I., Kudlai, O., Spraker, T.R., Tkach, V.V., Smales, L.R., Kuzmina, T.A., 2019. Morphological and molecular evidence for synonymy of *Corynosoma obtuscens* Lincicome, 1943 with *Corynosoma australe* Johnston, 1937 (Acanthocephala: Polymorphidae). Syst. Parasitol. 96, 95–110. https://doi.org/10.1007/s11230-018-9830-0

Sasaki, M., Katahira, H., Kobayashi, M., Kuramochi, T., Matsubara, H., Nakao, M., 2019. Infection status of commercial fish with cystacanth larvae of the genus *Corynosoma* (Acanthocephala: Polymorphidae) in Hokkaido, Japan. Int. J. Food Microbiol. 305, 108256. https://doi.org/10.1016/j.ijfoodmicro.2019.108256

Waindok, P., Lehnert, K., Siebert, U., Pawliczka, I., Strube, C., 2018. Prevalence and molecular characterisation of Acanthocephala in Pinnipedia of the North and Baltic Seas. Int. J. Parasitol. Parasites Wildl. 7, 34–43. https://doi.org/10.1016/j.ijppaw.2018.01.002

Table S3.1. Source data for the 61 *Corynosoma* sequences retrieved from GenBank that were used as additional data in the expanded TCS network analyses.

GenBank acc. no.	Species	Host species	Locality	Reference
EF467870	*C. strumosum*	*Phoca vitulina*	Monterey Bay, California, USA	García-Varela and Pérez-Ponce de León, 2008
EF467871	*C. strumosum*	*Phoca hispida botnica*	Baltic Sea, Finland	García-Varela and Pérez-Ponce de León, 2008
EF467872	*C. magdaleni*	*Phoca hispida saimensis*	Lake Saimaa, Finland	García-Varela and Pérez-Ponce de León, 2008
MF001277	*C. semerme*	*Halichoerus grypus*	Baltic Sea	Waindok et al., 2018
MF001278	*C. nortmeri*	*Phoca vitulina*	North Sea	Waindok et al., 2018
MF078642	*C. magdaleni*	*Phoca vitulina*	North Sea	Waindok et al., 2018
MK119250	*C. strumosum*	*Zalophus californianus*	Sausalito, California, USA	Lisitsyna et al., 2019
MK119253	*C. semerme*	*Callorhinus ursinus*	St. Paul Island, Alaska, USA	Lisitsyna et al., 2019
LC465307	*C. strumosum*	*Phocoena phocoena*	Rausu, Hokkaido, Japan	Sasaki et al., 2019
LC465308	*C. strumosum*	*Phocoena phocoena*	Rausu, Hokkaido, Japan	Sasaki et al., 2019
LC465309	*C. strumosum*	*Phocoena phocoena*	Rausu, Hokkaido, Japan	Sasaki et al., 2019
LC465310	*C. strumosum*	*Phocoena phocoena*	Rausu, Hokkaido, Japan	Sasaki et al., 2019
LC465311	*C. semerme*	*Phoca largha*	Abashiri, Hokkaido, Japan	Sasaki et al., 2019
LC465312	*C. semerme*	*Phoca largha*	Abashiri, Hokkaido, Japan	Sasaki et al., 2019
LC465313	*C. semerme*	*Phoca largha*	Abashiri, Hokkaido, Japan	Sasaki et al., 2019
LC465318	*C. strumosum*	*Phoca largha*	Abashiri, Hokkaido, Japan	Sasaki et al., 2019
LC465319	*C. strumosum*	*Phoca largha*	Abashiri, Hokkaido, Japan	Sasaki et al., 2019
LC465320	*C. strumosum*	*Phoca largha*	Abashiri, Hokkaido, Japan	Sasaki et al., 2019
LC465321	*C. strumosum*	*Phoca largha*	Abashiri, Hokkaido, Japan	Sasaki et al., 2019
LC465322	*C. strumosum*	*Osmerus dentex*	Akkeshi, Hokkaido, Japan	Sasaki et al., 2019
LC465323	*C. strumosum*	*Osmerus dentex*	Akkeshi, Hokkaido, Japan	Sasaki et al., 2019
LC465324	*C. strumosum*	*Osmerus dentex*	Akkeshi, Hokkaido, Japan	Sasaki et al., 2019
LC465325	*C. strumosum*	*Osmerus dentex*	Akkeshi, Hokkaido, Japan	Sasaki et al., 2019
LC465326	*C. strumosum*	*Osmerus dentex*	Akkeshi, Hokkaido, Japan	Sasaki et al., 2019
LC465327	*C. strumosum*	*Osmerus dentex*	Akkeshi, Hokkaido, Japan	Sasaki et al., 2019
LC465328	*C. strumosum*	*Clupea pallasii*	Nemuro, Hokkaido, Japan	Sasaki et al., 2019
LC465329	*C. strumosum*	*Clupea pallasii*	Nemuro, Hokkaido, Japan	Sasaki et al., 2019
LC465331	*C. strumosum*	*Osmerus dentex*	Nemuro, Hokkaido, Japan	Sasaki et al., 2019
LC465332	*C. strumosum*	*Osmerus dentex*	Akkeshi, Hokkaido, Japan	Sasaki et al., 2019
LC465342	*C. strumosum*	*Pleuronectes mochigarei*	Rumoi, Hokkaido, Japan	Sasaki et al., 2019
LC465345	*C. strumosum*	*Osmerus dentex*	Nemuro, Hokkaido, Japan	Sasaki et al., 2019
LC465346	*C. strumosum*	*Osmerus dentex*	Nemuro, Hokkaido, Japan	Sasaki et al., 2019
LC465347	*C. strumosum*	*Osmerus dentex*	Nemuro, Hokkaido, Japan	Sasaki et al., 2019
LC465348	*C. strumosum*	*Osmerus dentex*	Nemuro, Hokkaido, Japan	Sasaki et al., 2019
LC465352	*C. strumosum*	*Pleuronectes herzensteini*	Rausu, Hokkaido, Japan	Sasaki et al., 2019
LC465357	*C. strumosum*	*Osmerus dentex*	Nemuro, Hokkaido, Japan	Sasaki et al., 2019
LC465358	*C. strumosum*	*Osmerus dentex*	Nemuro, Hokkaido, Japan	Sasaki et al., 2019
LC465359	*C. strumosum*	*Osmerus dentex*	Nemuro, Hokkaido, Japan	Sasaki et al., 2019
LC465360	*C. strumosum*	*Osmerus dentex*	Nemuro, Hokkaido, Japan	Sasaki et al., 2019
LC465361	*C. strumosum*	*Osmerus dentex*	Abashiri, Hokkaido, Japan	Sasaki et al., 2019

LC465365	*C. strumosum*	*Myoxocephalus stelleri*	Abashiri, Hokkaido, Japan	Sasaki et al., 2019
LC465367	*C. strumosum*	*Myoxocephalus stelleri*	Abashiri, Hokkaido, Japan	Sasaki et al., 2019
LC465368	*C. strumosum*	*Pleuronectes obscurus*	Rausu, Hokkaido, Japan	Sasaki et al., 2019
LC465372	*C. strumosum*	*Pleuronectes herzensteini*	Rausu, Hokkaido, Japan	Sasaki et al., 2019
LC465373	*C. strumosum*	*Pleuronectes herzensteini*	Rausu, Hokkaido, Japan	Sasaki et al., 2019
LC465376	*C. strumosum*	*Pleurogrammus azonus*	Abashiri, Hokkaido, Japan	Sasaki et al., 2019
LC465377	*C. strumosum*	*Pleurogrammus azonus*	Abashiri, Hokkaido, Japan	Sasaki et al., 2019
LC465379	*C. strumosum*	*Sebastes trivittatus*	Nemuro, Hokkaido, Japan	Sasaki et al., 2019
LC465380	*C. strumosum*	*Hypomesus japonicus*	Akkeshi, Hokkaido, Japan	Sasaki et al., 2019
LC465381	*C. strumosum*	*Clupea pallasii*	Yubetsu, Hokkaido, Japan	Sasaki et al., 2019
LC465382	*C. strumosum*	*Clupea pallasii*	Yubetsu, Hokkaido, Japan	Sasaki et al., 2019
LC465384	*C. strumosum*	*Clupea pallasii*	Yubetsu, Hokkaido, Japan	Sasaki et al., 2019
LC465389	*C. strumosum*	*Hypomesus japonicus*	Nemuro, Hokkaido, Japan	Sasaki et al., 2019
LC465390	*C. strumosum*	*Hypomesus japonicus*	Nemuro, Hokkaido, Japan	Sasaki et al., 2019
LC465392	*C. semerme*	*Osmerus dentex*	Nemuro, Hokkaido, Japan	Sasaki et al., 2019
LC465394	*C. strumosum*	*Phoca vitulina*	Erimo, Hokkaido, Japan	Sasaki et al., 2019
LC465395	*C. strumosum*	*Phoca vitulina*	Erimo, Hokkaido, Japan	Sasaki et al., 2019
LC465399	*C. strumosum*	*Phoca largha*	Rebun, Hokkaido, Japan	Sasaki et al., 2019
LC465400	*C. strumosum*	*Phoca largha*	Rebun, Hokkaido, Japan	Sasaki et al., 2019
LC465401	*C. strumosum*	*Neophocaena phocaenoides*	Ibaraki, Japan	Sasaki et al., 2019
LC465402	*C. strumosum*	*Neophocaena phocaenoides*	Ibaraki, Japan	Sasaki et al., 2019

Table S3.2. Between- and within-group K2P distances among COI barcode sequences. Numbers represent means across all pairwise comparisons of sequences, with minimum and maximum values given in parentheses.

	C. strumosum	***C. semerme***	***Corynosoma* sp. 1**	***N***	**Within group**
C. strumosum				390	0.021 (0–0.051)
C. semerme	0.133 (0.115–0.151)			146	0.002 (0–0.014)
***Corynosoma* sp. 1**	0.056 (0.043–0.072)	0.131 (0.123–0.142)		4	0.007 (0–0.011)
***Corynosoma* sp. 2**	0.107 (0.078–0.119)	0.145 (0.134–0.153)	0.092 (0.074–0.098)	38	0.003 (0–0.021)

N – number of individuals

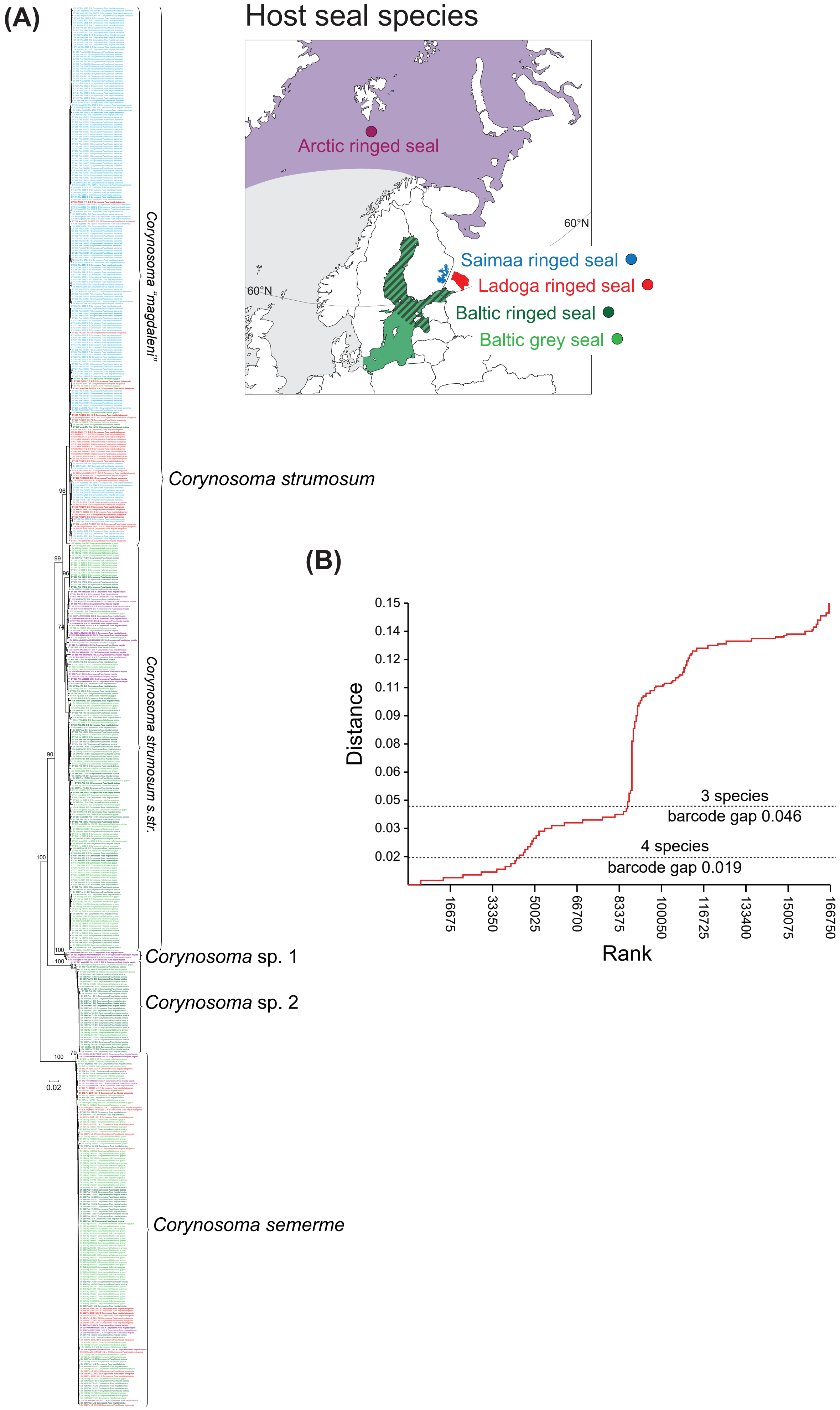
(A)
Host seal species
Arctic ringed seal
60°N
Saimaa ringed seal
Ladoga ringed seal
60°N
Baltic ringed seal
Baltic grey seal
Corynosoma "magdaleni"
Corynosoma strumosum
Corynosoma strumosum s.str.
Corynosoma sp. 1
Corynosoma sp. 2
Corynosoma semerme
96
99
96
75
90
100
100
100
100
79
0.02
(B)
Distance
0.15
0.14
0.12
0.11
0.09
0.08
0.06
0.05
0.03
0.02
3 species
barcode gap 0.046
4 species
barcode gap 0.019
16675
33350
50025
66700
83375
100050
116725
133400
150075
166750
Rank

Figure S3.1. (A) Neighbour-joining gene tree based on COI barcode sequences for 578 *Corynosoma* specimens. Individual names are coloured according to the seal (sub)species from which they originated (see legend), and include the specimen voucher code, host (sub)species abbreviation, seal individual code, and the intestinal section from which the individual was collected (SI – small intestine, CE – caecum, LI – large intestine). Within *C. strumosum* two clusters are indicated that had the highest percent identity to GenBank sequences of *C. magdaleni* and *C. strumosum*, respectively. Individuals that were used for RAD sequencing are shown in bold font. (B) A graphical summary of the ABGD species delimitation analysis. The plot shows ranked ordered pairwise distances calculated using the Kimura two-parameter (K2P) model. The dashed lines indicate two barcode gaps found for a prior maximal intraspecific divergence that ranges from 0.05 to 3.

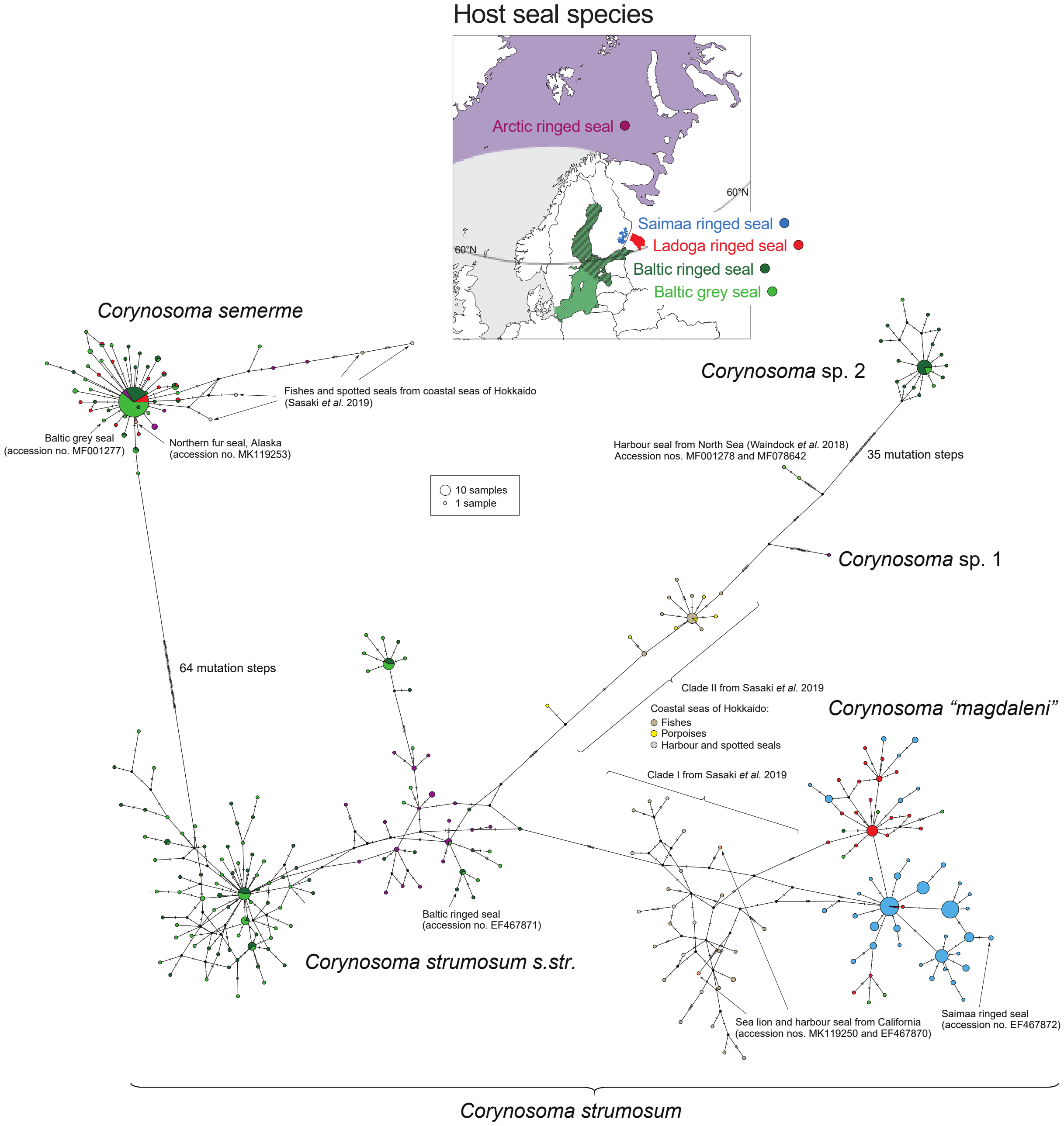

Figure S3.2. TCS haplotype network of *Corynosoma* COI barcode sequences showing the relationships among our recorded haplotypes ($N = 534$) and additional reference sequences retrieved from GenBank ($N = 61$). Circle and section colours denote host species, while the size of the circles is proportional to the number of haplotypes (see legend). Tick marks along branches denote mutational steps.
