# Supplementary material for: Loss of species and genetic diversity during colonization: Insights from acanthocephalan parasites in northern European seals
Source: Ecol Evol. 2023 Oct 19;13(10):e10608. doi: 10.1002/ece3.10608 (PMC10585441; doi:10.1002/ece3.10608)
Supplement: Supplementary file 4 — Appendix S4 [file ECE3-13-e10608-s001.pdf]

Appendix S4

3RAD library preparation

We generated RADseq libraries by implementing Design 2 of the Adapterama III 3RAD protocol (Bayona-Vásquez et al., 2019). For this 3RAD design, the genomic DNA was digested with MspI (Read 1 enzyme) and BamHI-HF (Read 2 enzyme), and ClaI was added as the third enzyme to suppress phosphorylated ends in MspI recognition sites and to avoid formation of adapter dimers.

We prepared two independently indexed libraries with 8 individuals included in in both as technical replicates. For this, 10 ng of genomic DNA from each individual was digested for 1 h at 37 °C in a solution with 1.5 µl of 10x Cutsmart® buffer (NEB®), 0.25 µl of MspI (NEB®) at 20 U/µl, 0.25 µl of BamHI-HF (NEB®) at 20 U/µl, 0.25 µl of ClaI at 20 U/ µl, 1 µl of i5Tru adapter at 2.5 µM, 1 µl of i7Tru adapter at 2.5 µM, and 0.75 µl of dH_2O. After digestion, we added 2.5 µl of H_2O, 1.5 µl ATP at 10 µM, 0.5 µl of 10x Ligase Buffer (NEB®), and 0.25 µl of T4 DNA Ligase at 400 U/µl to the same solution. The solution was incubated at 22 °C for 20 min and 37 °C for 10 min for two cycles, followed by a final cycle of 80 °C for 20 min. The i5 and i7 adapters (BadDNA, University of Georgia) were ligated to each sample using unique combinations of eight i5 and twelve i7 indexes. After digestion/ligation, samples were pooled and cleaned with 1.2x SeraMag SpeedBeads (Fisher Scientific™) in a 1.2:1 SpeedBeads:DNA ratio. Enrichment PCR of each library was carried with 10 µl of 5x Kapa Long Range Buffer (Kapa Biosystems, Inc.), 0.25 µl of KAPA LongRange DNA Polymerase at 5 U/µl, 1.5 µl of dNTPs mix (10 mM each dNTP), 3.5 µl of $MgCl_2$ at 25 mM, 2.5 µl of iTru5 primer at 5 µM, 2.5 µl of iTru7 primer at 5 µM, and 5 µl of pooled DNA. The temperature conditions for PCR enrichment were 94 °C for 2 min of initial denaturation, followed by 10 cycles of 94 °C for 20 sec, 57 °C for 15 sec and 72° for 30 sec, and a final cycle of 72 °C for 5 min. Two enriched libraries were each cleaned and quantified with a Quantus™ Fluorometer, pooled in library pool and sent to the Norwegian Sequencing Centre (University of Oslo, Norway) for quality control and subsequent final size selection using a one-sided bead clean-up (0.7:1 ratio) to capture 550 bp +/- 10% fragments. The library pool was sequenced in a 150 bp paired-end run on one lane of an Illumina HiSeq 4000 platform.

Details of bioinformatic analyses

RADseq reads were demultiplexed using the *process_radtags* module of the Stacks v.2.53 pipeline (Catchen et al., 2013) with the additional flags: -r (rescue barcodes and RAD-tags), -c (clean data) and -q (discard low-quality reads). Only intended RAD loci (generated by MspI and BamHI-HF enzymes) were included. Based on the number of retained sequences, one *Corynosoma* individual from a Ladoga

ringed seal that was not successfully sequenced (yielding less than 200,000 reads) was excluded from further analyses.

We optimized the clustering threshold by conducting exploratory analyses with four different values (0.8, 0.85, 0.9 and 0.95) and then comparing the number of loci recovered and the error rates between them. Locus and SNP error rates were calculated following Mastretta-Yanes et al. (2015). Locus error rate was defined as the number of loci present in only one of the samples of a replicate pair divided by the total number of loci found. SNP error rate was defined as the proportion of SNP mismatches between replicate pairs. We chose the value of 0.9 for the final assembly, because it gave the second-highest number of loci with low locus and SNP error rates (Figure S4.1).

To verify that replicated samples (sequenced in separate libraries) correctly grouped together, we calculated pairwise Euclidean distances among SNPs genotypes and constructed neighbor-joining trees in the ape v. 5.7 R package (Paradis and Schliep, 2019). Both samples of the eight replicate pairs clustered together regardless of the similarity threshold used (Figure S4.2). Reads from the replicated samples were then pooled for the final assembly.

For the final assembly, we used all ipyrad defaults with the following exceptions: ‘pairddrad’ for datatype, ‘filter based on quality scores’ for filter adapters, and ‘0.90’ for clustering threshold. The parameter ‘min samples per locus’ was set to ensure RAD locus sharing between COI barcode groups (Appendix S3) as follows: at least 13 individuals that matched the *C. magdaleni* GenBank sequence, 20 individuals of *C. strumosum s.str.* + *Corynosoma* sp. 1, 8 of *C. semerme*, and 5 of *Corynosoma* sp. 2. Such filtering resulted in an assembly of 1,125 RAD loci (Figure S4.3). These loci were then blasted against the NCBI nucleotide database, using BLAST 2.10 with an “*E*-value” significance threshold of 1×10^{-6}. Loci that had hits to mammalian sequences (indicative of potential contamination from the host) were discarded from further analysis.

References

Bayona-Vásquez, N.J., Glenn, T.C., Kieran, T.J., Pierson, T.W., Hoffberg, S.L., Scott, P.A., Bentley, K.E., Finger, J.W., Louha, S., Troendle, N., Diaz-Jaimes, P., Mauricio, R., Faircloth, B.C., 2019. Adapterama III: Quadruple-indexed, double/triple-enzyme RADseq libraries (2RAD/3RAD). PeerJ 7, e7724. https://doi.org/10.7717/peerj.7724

Catchen, J., Hohenlohe, P.A., Bassham, S., Amores, A., Cresko, W.A., 2013. Stacks: an analysis tool set for population genomics. Mol. Ecol. 22, 3124–3140. https://doi.org/10.1111/mec.12354

Mastretta-Yanes, A., Arrigo, N., Alvarez, N., Jorgensen, T.H., Piñero, D., Emerson, B.C., 2015. Restriction site-associated DNA sequencing, genotyping error estimation and de novo assembly

optimization for population genetic inference. Mol. Ecol. Resour. 15, 28–41. https://doi.org/10.1111/1755-0998.12291

Paradis, E., Schliep, K., 2019. ape 5.0: an environment for modern phylogenetics and evolutionary analyses in R. Bioinformatics 35, 526–528. https://doi.org/10.1093/bioinformatics/bty633

Table S4.1 Individual-specific results after filtering and clustering of RAD sequences for 91 focal *Corynosoma* specimens and 11 outgroup individuals.

Sample ID	No. raw reads	Clusters at 90%	No. consensus loci	No. loci in assembly
**EY_004**	2373247	65413	62183	930
**EY_013**	4427254	167519	160894	466
**EY_015**	1857734	55073	52060	910
**EY_016**	4034865	107526	103325	969
**EY_020**	1138756	23463	22119	840
**EY_031**	3473885	145406	141178	596
**EY_048**	364732	7467	6738	90
**EY_051**	3168239	81904	77981	971
**EY_057**	2586700	69659	66326	949
**EY_059**	2661278	130035	126152	635
**EY_061**	4307792	147916	143385	961
**EY_064**	5468646	49985	46355	934
**EY_087**	4497837	155857	150440	954
**EY_092**	3203609	136091	132262	353
**EY_093**	3738817	149978	146035	728
**EY_101**	2963968	54200	51200	946
**EY_107**	3004696	116823	112657	922
**EY_109**	2593705	67317	63743	936
**EY_110**	1651425	67341	64563	865
**EY_133**	1988730	34410	32342	929
**EY_134**	2241125	31891	29564	912
**EY_141**	3150283	30232	28176	906
**EY_142**	3249550	32686	30685	917
**EY_149**	2963588	134716	130892	801
**EY_151**	1933530	75185	72402	926
**EY_161**	3553199	117280	112939	965
**EY_164**	1549538	25947	24297	859
**EY_174**	2459548	94161	90932	935
**EY_179**	2997082	82073	78071	982
**EY_183**	2770514	38477	36257	917
**EY_211**	2342945	34765	32632	878
**EY_217**	3714387	72645	68982	970
**EY_235**	2051298	111837	108699	405
**EY_240**	2186554	36917	34259	892
**EY_249**	7900053	196771	188770	861
**EY_258**	2362022	36695	34641	933
**EY_260**	2259864	91450	88004	845
**EY_288**	3344367	33462	31415	916
**EY_337**	991367	22585	21254	825
**EY_347**	2172039	28440	26676	909
**EY_357**	3551887	34851	32498	914
**EY_359**	6997516	46129	42431	899
**EY_365**	3261466	35403	33173	918
**EY_367**	2548221	34988	33027	951

**EY_372**	1244009	24341	22913	871
**EY_374**	929927	49211	47367	307
**EY_393**	1276563	45265	42825	834
**EY_401**	2874960	130456	126619	874
**EY_402**	2106647	33500	31504	915
**EY_414**	1075755	22647	21268	841
**EY_427**	2586597	71686	67868	920
**EY_464**	2890427	30830	29052	909
**EY_469**	2552422	40016	37378	906
**EY_479**	2474027	38327	36091	963
**EY_487**	2604119	125476	121773	754
**EY_490**	2839107	125272	121925	110
**EY_492**	2138998	112776	109312	614
**EY_493**	2868204	127295	123547	300
**EY_494**	1840286	98825	96137	84
**EY_497**	1336798	47442	45081	799
**EY_499**	1354854	75648	72623	524
**EY_500**	514590	16483	15260	450
**EY_501**	1392257	47907	45057	839
**EY_504**	1045450	25572	24018	832
**EY_506**	1692684	91147	87910	714
**EY_509**	1824381	54903	52442	917
**EY_513**	3249375	90011	86026	947
**EY_522**	2216349	28988	27407	897
**EY_530**	3116826	54224	51365	932
**EY_536**	2380913	50458	47519	924
**EY_546**	1805939	28775	27073	915
**EY_547**	1588338	58610	55593	785
**EY_548**	1242661	56592	54099	753
**EY_549**	3424361	91911	87537	890
**EY_550**	1931627	48143	45631	934
**EY_554**	3727137	149335	144848	611
**EY_556**	2301092	117616	114002	789
**EY_557**	2338094	35333	33034	909
**EY_558**	2363313	102576	98798	921
**EY_560**	860777	27669	26106	710
**EY_562**	4114981	35888	33612	913
**EY_567**	2743285	35942	33620	856
**EY_569**	2717033	33351	31241	924
**EY_572**	1888355	30114	28237	913
**EY_574**	2567735	59228	56085	966
**EY_576**	2896393	32968	30431	913
**EY_578**	3287545	34502	31784	898
**EY_579**	3561521	137615	131106	901
**EY_581**	2470075	122792	119090	550
**EY_588**	965870	38164	36016	719
**EY_590**	3697789	145403	140914	482
**LS_064**	477848	14124	12643	129

**LS_073**	875260	24920	22910	163
**LS_075**	1763973	31122	29123	189
**LS_076**	1449251	31760	29309	176
**LS_195**	2533780	39629	36102	188
**LS_207**	2476037	42470	39403	335
**LS_208**	1119267	30220	28072	179
**LS_209**	888565	25364	22903	159
**LS_212**	2763113	41647	38818	207
**LS_213**	1575678	29677	27212	194
**LS_214**	2405041	41063	37303	233

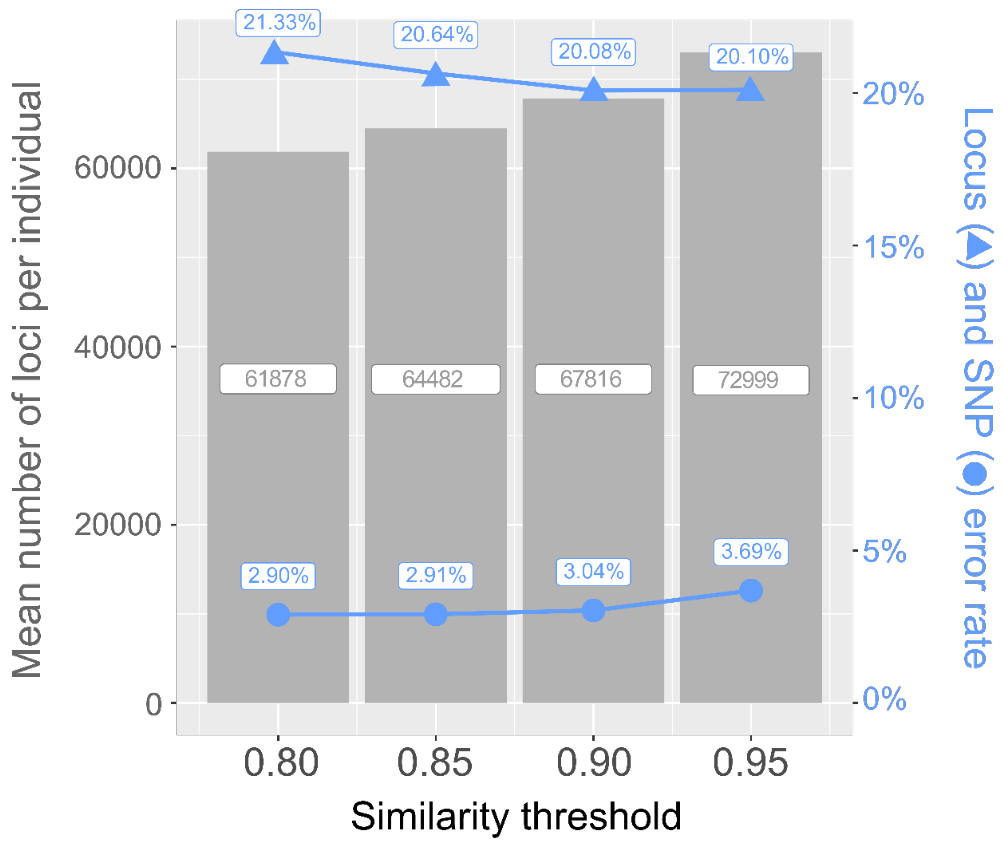

Figure S4.1. Locus and SNP error rates and mean number of RAD loci recovered per individual for the four tested clustering thresholds.

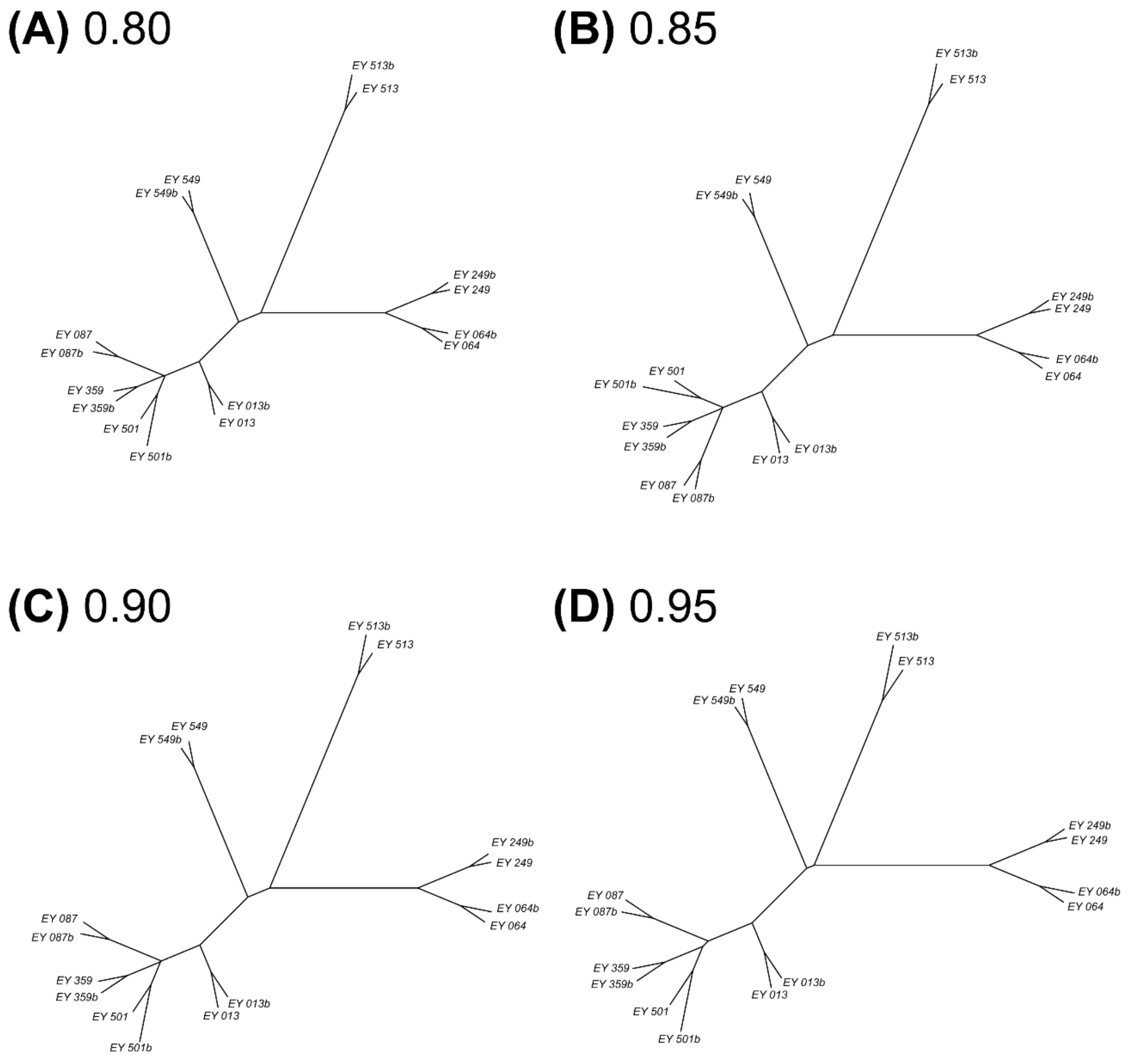

Figure S4.2. Neighbor-joining trees based on Euclidean distances among vectors of alleles created using different clustering thresholds (A–D) for *de novo* assembly of RAD loci.

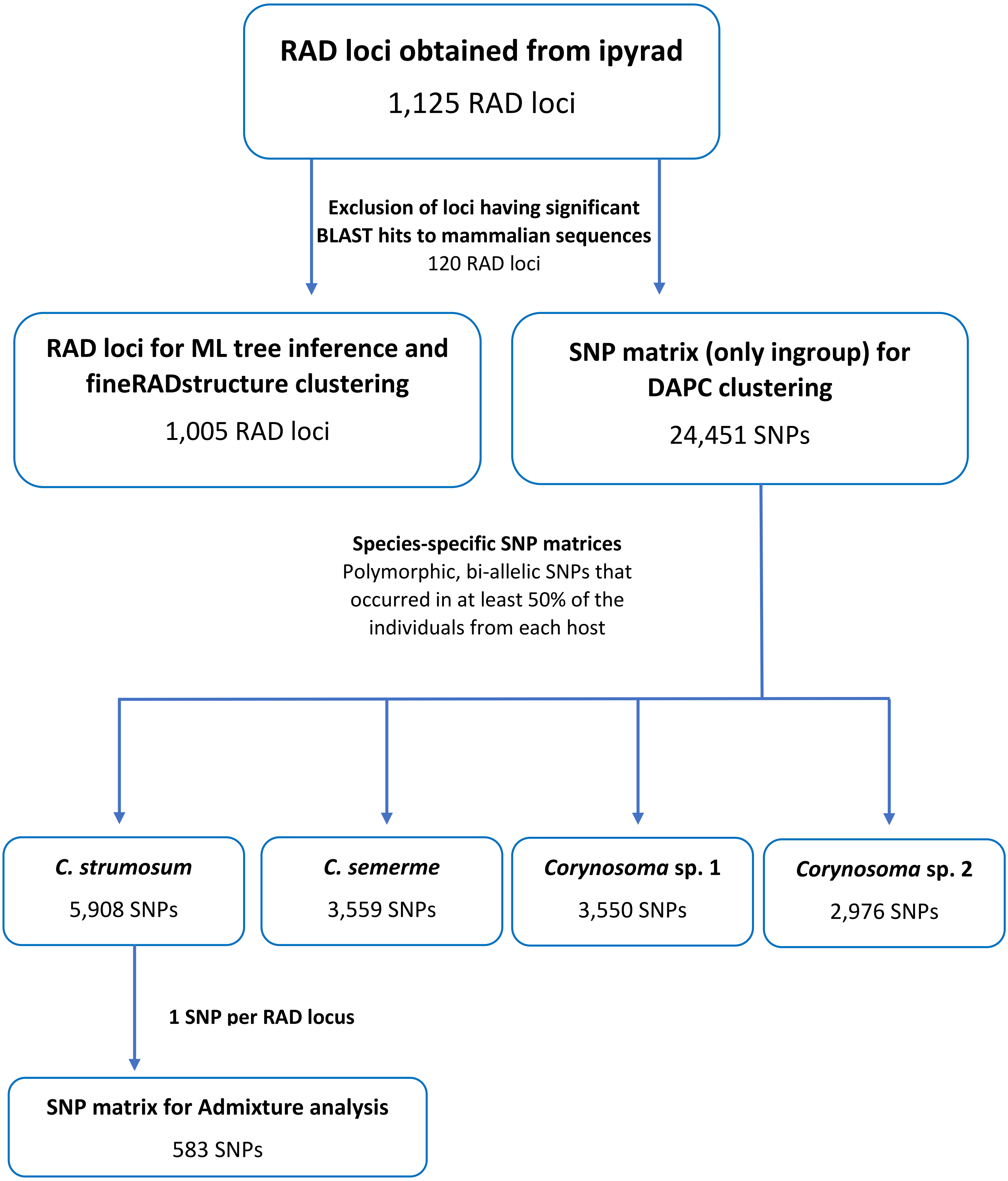

Figure S4.3. Schematic representation of the construction of the different RADseq datasets used in the different analyses.

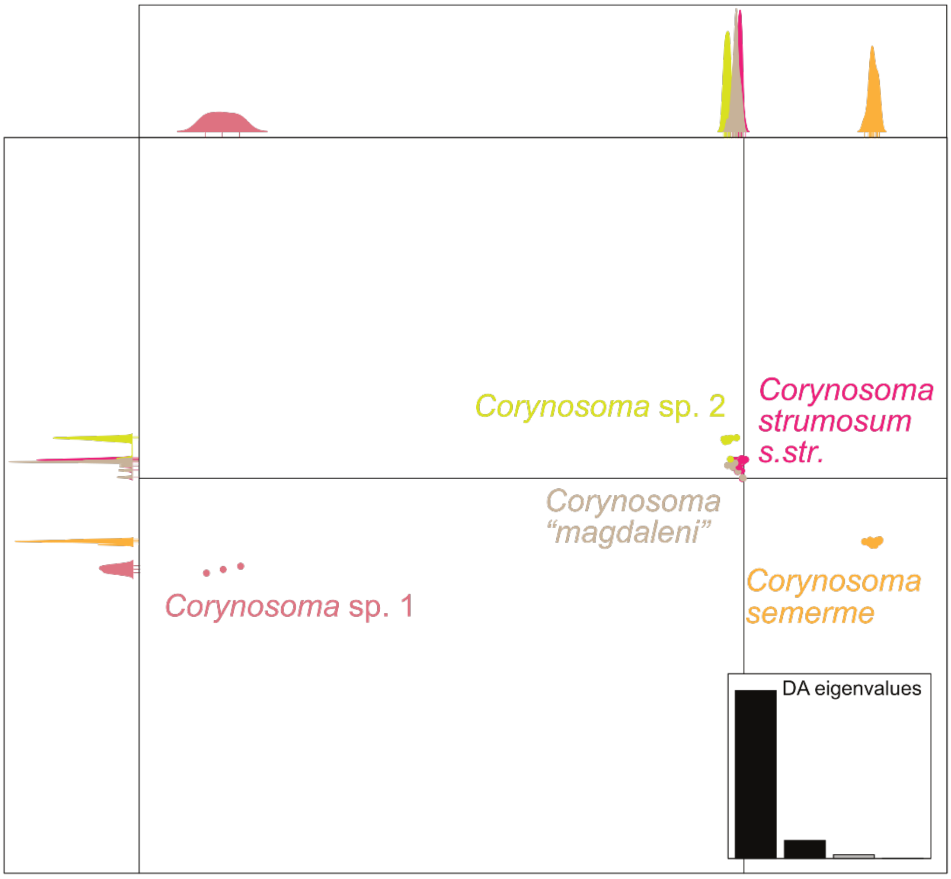

Figure S4.4. Discriminant Analysis of Principal Components (DAPC) scatter plot for 91 *Corynosoma* individuals based on 24,451 SNPs obtained with a priori grouping corresponding to the five main clusters of the COI barcode tree (Figure S2.1). Distributions along the top and left of the scatter plot represent the densities of individuals along a given discriminant function. For the analysis, the 10 first PCs were retained, which accounted for 85.4% of the variance.

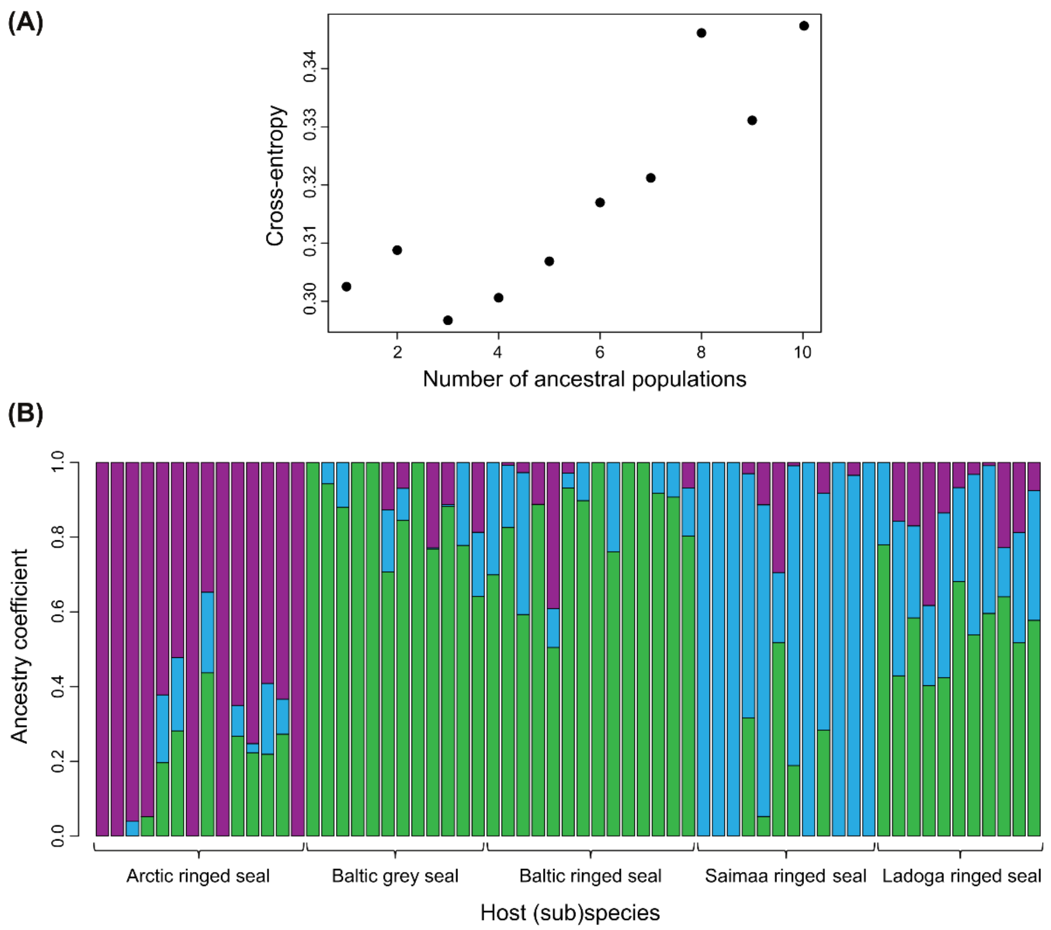

Figure S4.5. Estimation of population-genetic structure within *Corynosoma strumosum* based on RADseq data. (A) Minimal cross-entropy for different numbers of ancestral populations. (B) Plots of posterior probabilities of assignment for each individual to each of the three proposed ancestral populations. Each vertical bar represents an individual, and colours denote the three different ancestral populations. The seal host (sub)species from which each individual was collected is indicated below the bar plot.
